# How the individual human mobility spatio-temporally shapes the disease transmission dynamics

**DOI:** 10.1038/s41598-020-68230-9

**Published:** 2020-07-09

**Authors:** Suttikiat Changruenngam, Dominique J. Bicout, Charin Modchang

**Affiliations:** 1 Biophysics Group, Department of Physics, Faculty of Science, Mahidol University, Bangkok, 10400 Thailand; 2 Institut Laue-Langevin, 71 Avenue des Martyrs, 38042 Grenoble, France; 3 EPSP, TIMC Laboratory, UMR CNRS 5525, Grenoble Alpes University, VetAgro Sup, Grenoble, France; 4 Centre of Excellence in Mathematics, CHE, Bangkok, 10400 Thailand; 5 Thailand Center of Excellence in Physics, CHE, 328 Si Ayutthaya Road, Bangkok, 10400 Thailand

**Keywords:** Applied physics, Biological physics, Statistical physics, thermodynamics and nonlinear dynamics

## Abstract

Human mobility plays a crucial role in the temporal and spatial spreading of infectious diseases. During the past few decades, researchers have been extensively investigating how human mobility affects the propagation of diseases. However, the mechanism of human mobility shaping the spread of epidemics is still elusive. Here we examined the impact of human mobility on the infectious disease spread by developing the individual-based SEIR model that incorporates a model of human mobility. We considered the spread of human influenza in two contrasting countries, namely, Belgium and Martinique, as case studies, to assess the specific roles of human mobility on infection propagation. We found that our model can provide a geo-temporal spreading pattern of the epidemics that cannot be captured by a traditional homogenous epidemic model. The disease has a tendency to jump to high populated urban areas before spreading to more rural areas and then subsequently spread to all neighboring locations. This heterogeneous spread of the infection can be captured by the time of the first arrival of the infection $(T_{fi} )$, which relates to the landscape of the human mobility characterized by the relative attractiveness. These findings can provide insights to better understand and forecast the disease spreading.

## Introduction

Over the past several decades, outbreaks of emerging infectious diseases have been occurring at an increasing rate^1^. They have a considerable impact not only on public health and health care but also on various forms of social and economic well-being^2^. However, despite the increased attention on infectious diseases, there is still a lack of comprehensive knowledge of mechanisms controlling the spatiotemporal propagation of infectious diseases. Traditional homogenous epidemic models, e.g., a compartmental epidemic model, have long been helpful in understanding the foundation of transmission dynamics of infectious diseases. These homogenous epidemic models assume all human individuals to be well-mixed and ignore heterogeneity that may be resulted from, for example, ages, locations, and contact patterns of human individuals. Although these homogeneous models are useful in certain situations, in order to gain more accurate modelling results, incorporating factors describing spatiotemporal heterogeneity in disease transmission dynamics into the epidemic models may be necessary.

In reality, the transmission dynamics of real-world infectious diseases are governed by several spatiotemporal heterogeneous factors, including the seasonality of diseases^3^, the contact networks of the host population^4^, population heterogeneity^5^, and human mobility^6^. Human mobility, in particular, undeniably plays a crucial role in the temporal and spatial transmission dynamics of infectious diseases. During the past few decades, the inclusion of the human mobility mechanism in the epidemic models has been considered in many studies for modeling of the geographical spread of diseases^7–14^. Human traveling not only has a significant effect on the epidemic spreading^15–17^, it also remarkably influences the speed of disease spreading^7,8,15^. Moreover, understanding the roles of human mobility on disease spreading is notably useful for designing effective disease control strategies, such as vaccination or travel restriction^18–22^.

Due to the approachability of human travelling data (e.g., check-ins from locations, GPS navigators, or mobile-phone records), scientists have discovered several properties and characteristics of human mobility^23–29^. A myriad of human mobility models has been developed to understand the fundamental mechanism hidden behind these findings^25,30–32^. These human mobility models are becoming more and more accurate, capable of reproducing human mobility either at an individual level^25^ or at collective information^31^. Although several research works have studied the effects of human mobility on the spread of diseases^33–36^, incorporating a realistic individual human mobility model into an epidemic model is still needed. Integrating an epidemic model with an individual human mobility model is necessary for investigating how an individual infectious person geo-temporally spread the disease.

In this article, we address the question of how individual human mobility spatiotemporally shapes the spread of communicable infections. To this end, we integrate a classical SEIR (susceptible, exposed, infectious and recovered) individual-based model with an individual human mobility model proposed by Yan et al.^37^, both at the individual and population level, where the transition probabilities between localities are governed by both a gravity-like part and a memory part. To follow the spread of the infection, we have introduced an indicator, “the time of the first arrival of the infection ($$T_{fi} )$$”, so that an infection which started at an origin point reaches another given point for the first time. To characterize the landscape of human mobility, we also defined the “relative attractiveness (RA)” based on a transition matrix between localities. Now, our objective of investigating how individual human mobility shapes the spread amounts to determining or constructing the relationship between $$T_{fi}$$ and the explanatory variable, RA. Indeed, the quantity $$T_{fi}$$ can not only elucidate the direction of epidemics driven by human mobility but also provide the velocity of the spreading by calculating its spatial derivative in the RA space. As an illustrative application, we considered the spread of human influenza in two contrasting countries, namely, Belgium and Martinique, as case studies, to assess the specific roles of human mobility on infection propagation. Interestingly, we found in both cases that $$T_{fi}$$ follows a truncated power law as a function of RA, indicating a one-to-one relationship between spatiotemporal infection spread and host mobility landscape.

## Results

### Individual human mobility-driven disease transmission dynamics

**Figure 1:**
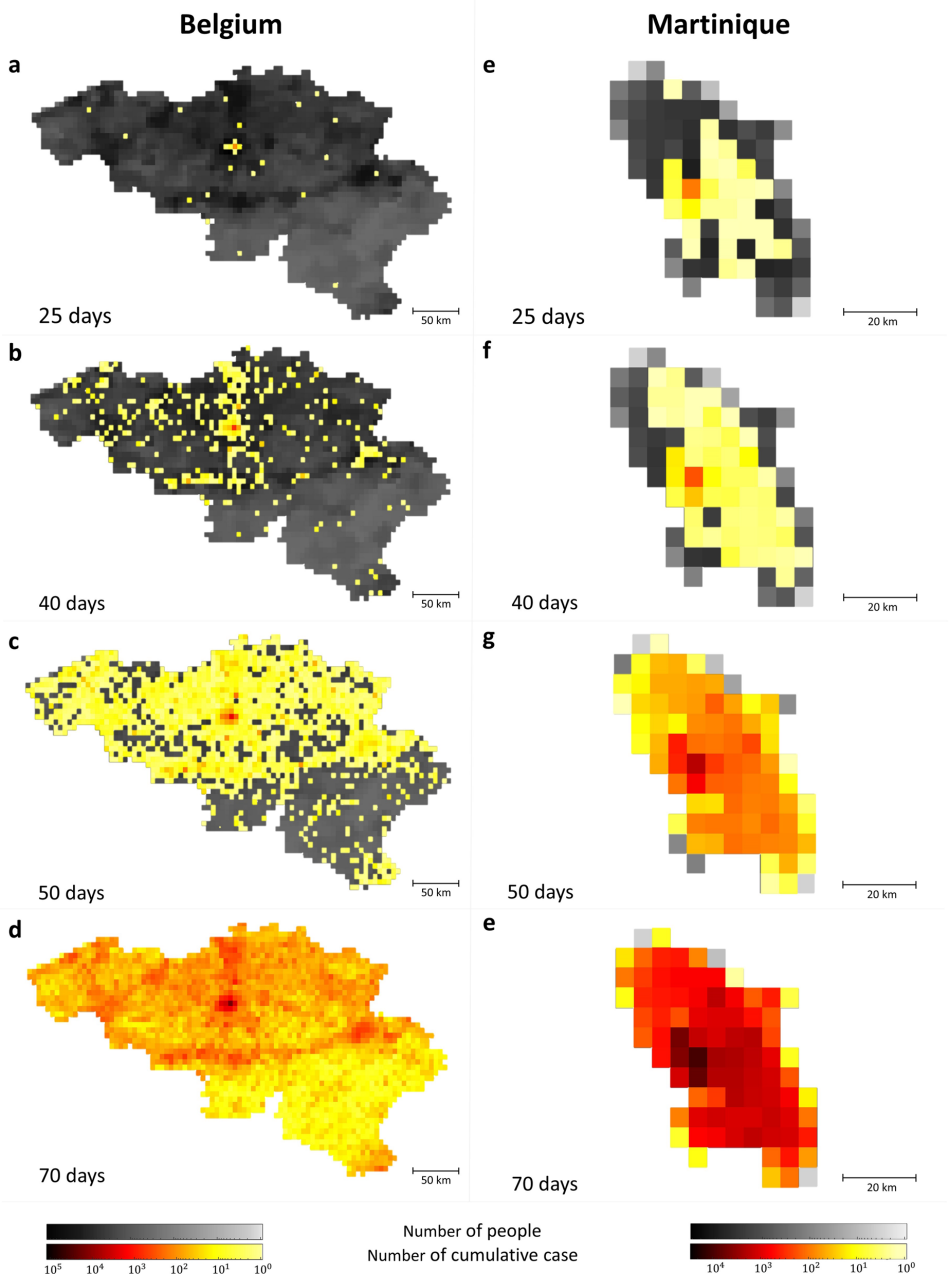
Geotemporal spreading pattern of an uncontrolled epidemic in Belgium and Martinique. The snapshots show a spreading pattern of an epidemic in Belgium (left column) and Martinique (right column) with *R*_0_ = 1.9 at 25, 40, 50, and 70 days after the introduction of the disease. The shade of grey represents the population density, and the shade of red indicates the cumulative number of infected individuals in each location. Population sizes simulated for Belgium and Martinique are $1.1 \times 10^{7}$ within 2,252 locations and $4 \times 10^{5}$ within 86 locations, respectively. The parameter values used in the simulations are summarized in Table 1 in the Methods section.

A geo-temporal spreading pattern of an epidemic in Belgium and Martinique is illustrated in Fig. 1. At the beginning of the simulation, there is only one symptomatic infectious individual at the most populated location (red dot in Fig. 1), while all other individuals are susceptible to the disease. Each individual stays at a particular location for a certain period of time, ($\Delta T$), following the waiting time distribution with the average waiting time $\left\langle {\Delta T} \right\rangle = 5$ days. We found that at the early time of the epidemic, the disease is likely to reach urban areas with high population densities and after an intermediate time (e.g., at day 50), it spreads to rural areas with lower population densities. Lastly, at the late time, the disease is gradually transmitted to all neighboring locations and reaches the boundary of the country. However, the epidemic in Belgium seems more dispersed than in Martinique, which might be due to the fact that the population distribution in Belgium is more heterogeneous than in Martinique.

**Figure 2:**
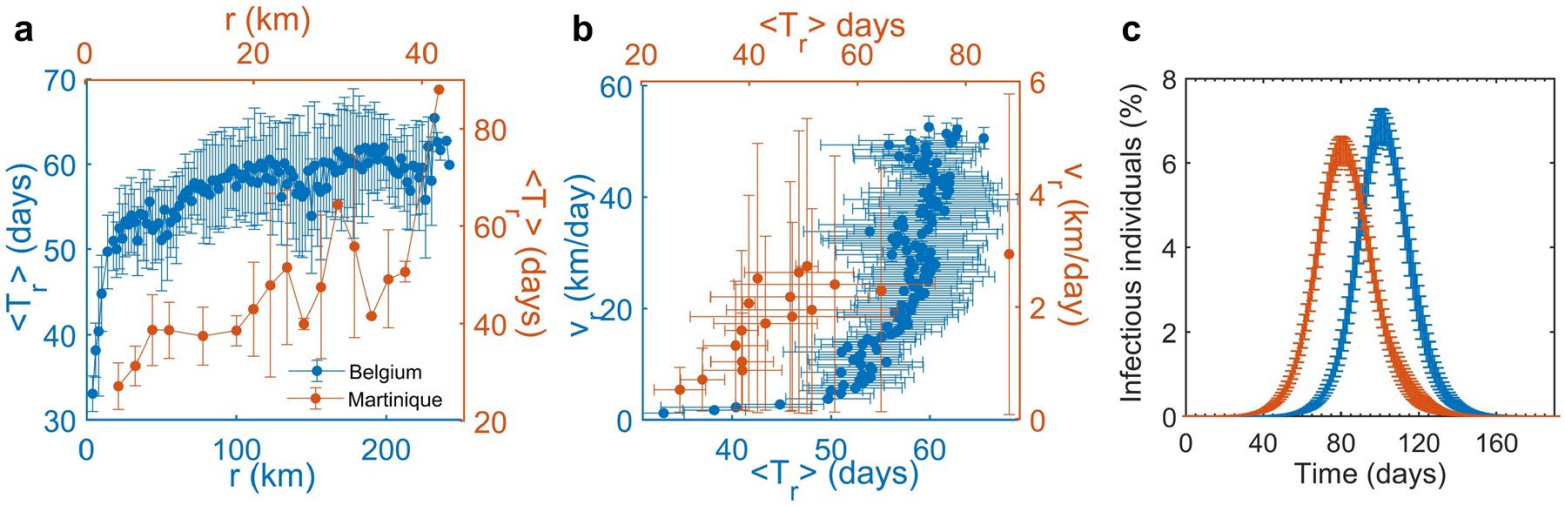
Radial speed of disease spread. (**a**) The radial-averaged time of the first arrival ($\left\langle {T_{r} } \right\rangle$) of infection in each location at radius $r$ centered at the first infected location for Belgium (blue) and Martinique (red) and **(b)** the corresponding radial speed of spread. (**c**) The epidemic curves showing the number of infectious individuals in Belgium (blue) and Martinique (red).

To have an idea of how fast the disease spatially spreads, we computed the radial-averaged time of the first arrival ($\left\langle {T_{r} } \right\rangle$) of infection in each location (Fig. 2a) and the radial speed of spread at radius $r$ centered at the first infected location (Fig. 2b). At the early time of the epidemic, the disease spreads locally and slowly, spending time around 50 days to reach 15 km in Belgium (Blue line) and around 40 days to reach 10 km in Martinique (red line). In contrast, after some periods of time, the disease spread faster and faster due to the jumping of infectious individuals to distant locations (Fig. 2b). Note, however, that, although the disease spatially spreads faster in Belgium, the epidemic curve showing the percentage of infectious individuals in Belgium increases slower than that in Martinique (Fig. 2c). This might be because Belgium is bigger, and the population distribution in Belgium is more heterogeneous than that in Martinique. Fluctuations in $\left\langle {T_{r} } \right\rangle$ indicate that different locations at similar *r* have different mobility, causing the variety of the arrival time of infectious individuals (Fig. 2a). Moreover, when considering the effect of the averaged waiting time on the radial speed of disease spreading, we found that increasing the averaged waiting time affects the speed of the spatial spreading of disease in Belgium more than in Martinique (see Figure S4).

**Figure 3:**
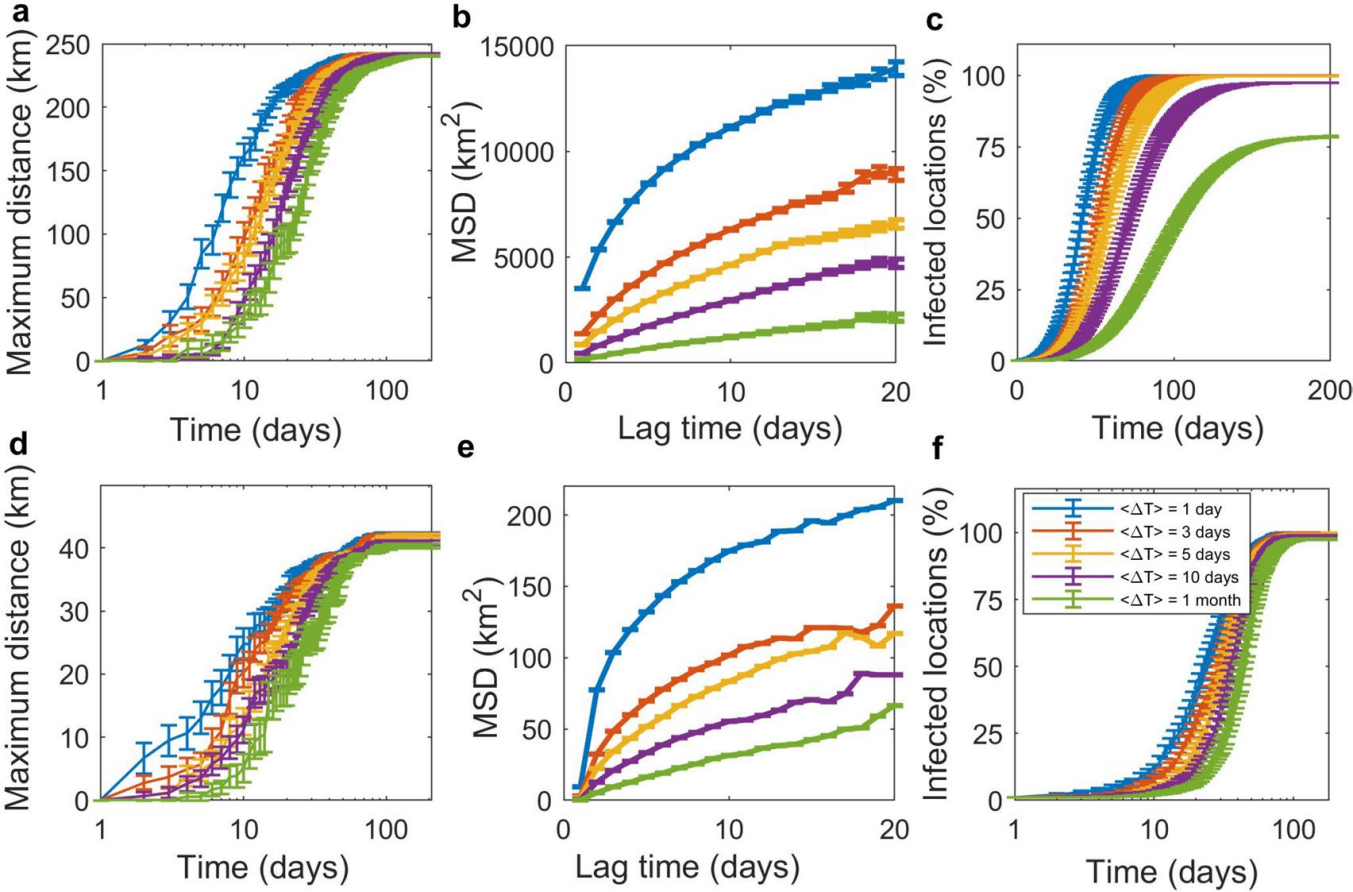
Effects of waiting time on the disease spreading characteristics. (**a**, **d**) The maximum distance, which is the distance between the first infected location and the farthest infected location. (**b**, **e**) The time-averaged mean square displacement (MSD) of infectious individuals as a function of lag time. (**c**, **f**) The cumulative number of infected locations. The top and bottom rows show the simulation results for Belgium (with the population density reduced by 100 times) and Martinique, respectively. The number of simulations for Belgium and Martinique was 50 and 30, respectively.

In addition, the proposed model allows us to explore the movement behaviors of infectious individuals by tracking their movement trajectories. We elucidated the disease dispersal by computing the maximum distance of disease transmission, the time-averaged mean square displacement (MSD) of infectious individuals, and the number of infected locations (Fig. 3) for both Belgium and Martinique. However, since tracking the movement trajectories of the vast population requires a large amount of computer memory; therefore, the location and movement tracking of infectious individuals in Belgium was performed only in the case of the population size is reduced by 100 times. We have checked that after scaling down the population density in Belgium, there was no location with a zero population. The effects of scaling down the population density are discussed in the supplementary information. Also, to investigate the effects of waiting time on the dynamics of disease transmission, the model was simulated using a different value of waiting time ($\left\langle {\Delta T} \right\rangle$ = 1, 3, 5, 10, and 30 days). The maximum distance of disease transmission was measured from the radius between the first infected location and the farthest infected location at a certain time (Fig. 3a,d). We found that the disease initially spreads locally around the first infected location and then spreads rapidly and widely to other locations until it reaches the locations at the boundary with the maximum distance of 243 km and 42 km for Belgium and Martinique, respectively. Figure 3b, e show the MSD, $\left\langle {\delta^{2} (\Delta )} \right\rangle$, of infectious individuals as a function of lag time (Δ). Note that the MSD of infectious individuals could be measured only during the first 20 days after they become infectious. This is because the probability that an infectious individual will get recover after time Δ since they become infectious decays as $1 - \exp \left( { - \gamma \Delta } \right)$ , where $\gamma$ = 1/(3 days); therefore, less than 0.2% of infectious individuals are infectious longer than 20 days. Finally, according to Fig. 3c, f, the increasing number of infected locations is consistent with the increase of the maximum distance of disease transmission shown in Fig. 3a, d. However, due to a higher heterogeneity in the population distribution in Belgium, in the case of the averaged waiting time of 30 days, at day 150, only 79% of locations in Belgium are infected while almost 100% of locations in Martinique are infected (see also the epidemic curves in Figures S11a, S12a).

**Figure 4:**
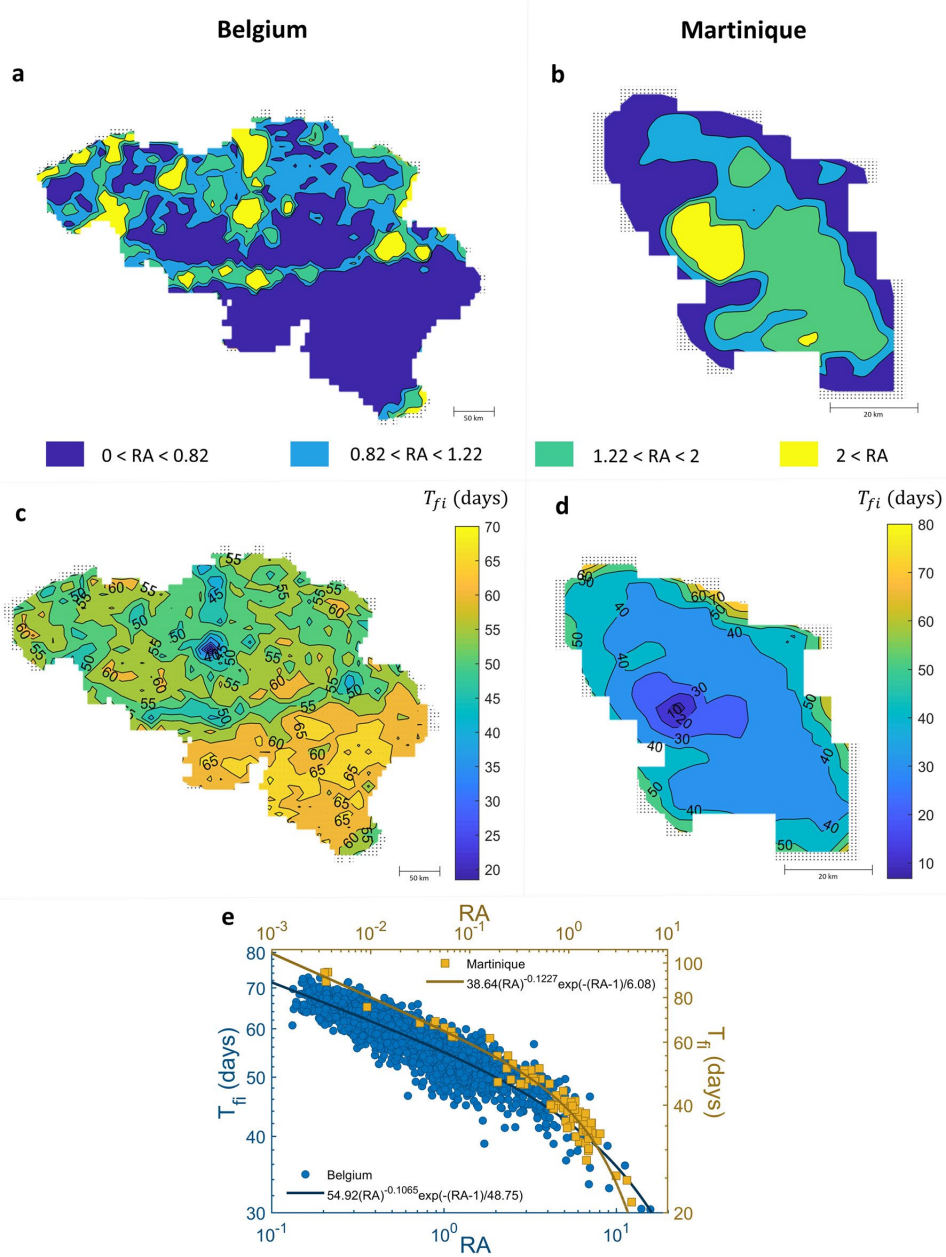
Relationship of the relative attractiveness of locations (RA) and disease spreading in Belgium and Martinique. (**a**, **b**) The maps illustrate the RA of each location in Belgium and Martinique, respectively. Each section represents the different RA with different colors. (**c**, **d**) The contours show the first arrival time ($T_{fi}$) of disease occurrence in each location after the end of epidemics; 6 months. (**e**) The relationship between RA and $T_{fi}$ of Belgium (blue) and Martinique (red) are fitted by the truncated power law.

The relative attractiveness (RA) was introduced to characterize the human mobility landscape. The RA maps for Belgium and Martinique are shown in Fig. 4a, b, respectively. We found that the RA is proportional to the population size in each location (Figure S7a, b), and the distribution of RA in Martinique seems more homogeneous than in Belgium (Figure S7c). The RA was classified into four groups with different colors; 0 < RA < 0.82, 0.82 < RA < 1.22, 1.22 < RA < 2, and RA > 2 representing low, medium, high, and very high relative attractiveness of locations, respectively. As can be seen in the RA maps, the locations with very high RA are more scattered in Belgium than in Martinique, and the locations with low RA are in a greater proportion in Belgium than in Martinique. Interestingly, for both Belgium and Martinique, there are approximately six locations in which the RA is comparatively very high (Figure S7d). Moreover, we found that the average RA of those six locations is three times greater in Belgium than in Martinique. Therefore, in Belgium, individuals have a tendency to be trapped in only a few particular locations in contrast to Martinique, where individuals are likely to visit more various locations.

To examine how human mobility shapes the disease spreading, RA is compared with the contour of the first arrival time ($T_{fi}$) of disease occurrence in each location, as shown in Fig. 4c for Belgium and Fig. 4d for Martinique. The contours show that the disease frequently jumps to an area with high RA and then spread into a lower RA area. Interestingly, the relationship between RA and $T_{fi}$ (Fig. 4e) follows a truncated power law as, $T_{fi} = t_{1} RA^{ - \nu } {\exp}\left( { - \left( {RA - 1} \right)/RA_{cut{-}off} } \right)$, where $t_{1}$ represents the arrival time that disease reaches on “flat lands” defined as locations where RA = 1, $\nu$ and $RA_{cut{-}off}$ describes the distribution of $T_{fi}$ for each location. Due to a more homogeneity of RA in Martinique, the disease is likely to spatially spread faster than in Belgium (Fig. 2b). Flat lands, especially, are reached by the disease around 39 days in Martinique and around 55 days in Belgium after the first infectious individual was introduced (see the fitted equations in Fig. 4e).

In addition, we also investigated the effect of waiting time and the population density on the relation between RA and $T_{fi}$. As, in our model, the human mobility and the epidemiological dynamics are deeply integrated, changing the population density will simultaneously affect both of them. We found that both factors significantly change the relationship in Belgium (Figures S8, S11) but not in Martinique (Figures S9, S12). All fit parameter values are shown in Figures S10 and S13. Besides RA, to understand how infectious individuals move, we measured the infectious-individual visitation frequency of a location by counting the number of times that infectious individuals visit a location. We found that the pattern of the visitation frequency map (Figure S5) is similar to the pattern of the RA map. Moreover, we calculated the probability distribution of the number of locations visited by infectious individuals, *P*(*S*), and the probability distribution of the number of step lengths among the visited locations of infectious individuals shown in Figure S6.

### Effects of restriction on symptomatic infectious individual movement

**Figure 5:**
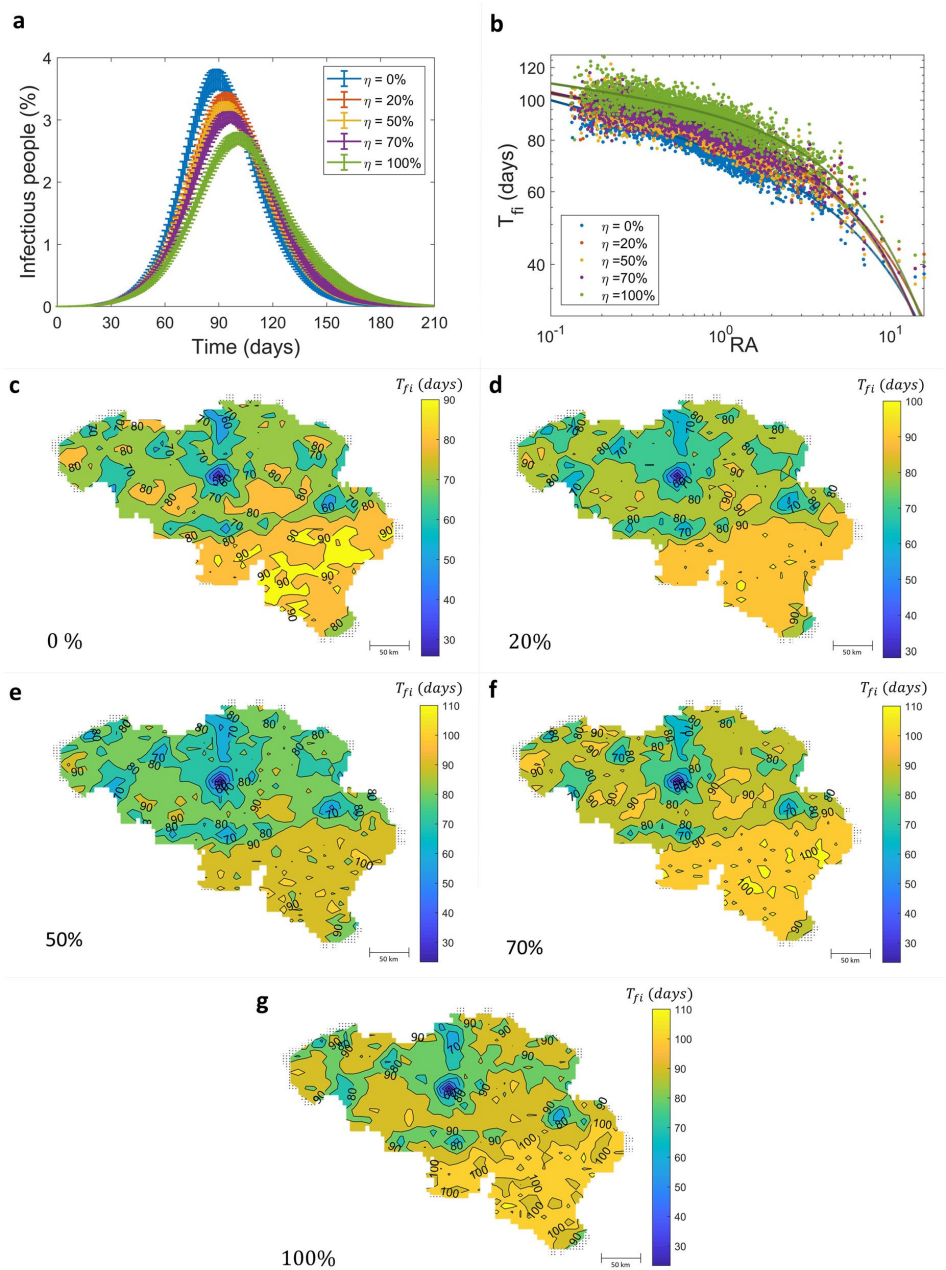
Effectiveness of symptomatic infectious individual movement restriction in Belgium. (**a**) The numbers of infectious individuals. (**b**) The relationship between RA and $T_{fi}$. Symbols indicate the results obtained from the model simulations. Lines correspond to the truncated power-law equation. (**c**–**g**) Contours showing the first arrival time of disease occurrence in each location with a different percentage of infectious individuals which are banned from travelling to other locations (*η*); 0%, 20%, 50%, 70%, and 100%, respectively.

Our model allows us to investigate disease control strategies at the individual human level. In the present work, the model was employed to investigate the effectiveness of restriction of infectious individual movements in Belgium. To reduce the time required for simulating the model, the simulations presented in this section were performed only in the case of the population size of Belgium is reduced by 100 times. The effects of scaling down the population size are discussed in the supplementary information. In practice, only symptomatic infectious individuals may be able to be identified, hence in our model, only (some) symptomatic infectious individuals are banned from traveling between locations while asymptomatic infectious individuals are free to travel according to the human mobility mechanism. Note that although the travel-restricted symptomatic infectious individuals cannot transmit the infection to other locations, they can transmit the disease within the locations that they are staying while they are infectious. The time course of the numbers of infectious individuals at a different percentage of symptomatic infectious individuals restricted to travel to other locations, $\eta$, are shown in Fig. 5a. The numbers of infectious individuals decrease significantly when the restriction percentage increases. For 100% travel restriction (green curve), it could delay the epidemic peak from day 89 to day 101 and could also reduce the epidemic size from 65.22 to 57.85%. Figure 5b shows a change in the relation between RA and $T_{fi}$, in which all fit parameter values are shown in Figure S14. The disease spreads slower when a higher number of symptomatic infectious individuals is banned from travelling; the arrival time of disease reaching on flat lands, $t_{1}$, is 78, 86, 88, 90, and 97 days for $\eta = 0\%$, $20\%$, $50\%$, $70\%$, and $100\%$, respectively. All contours of $T_{fi}$ with different $\eta$ are shown in Fig. 5c–g.

## Discussion and conclusions

Of several factors, human mobility is an important factor affecting the spread of epidemics. Many researchers have been extensively investigating the role of human mobility on the spatial spread of influenza-like illnesses (ILIs) and other emerging infectious diseases^6,9,10,13,18,38–40^. However, how the infection or the transmission chain would spread and spread with what associated speeds due to human mobility remains to be a challenging question. Therefore, to deeply understand the impact of human mobility on the epidemics, we exploited a classical SEIR individual-based model that integrates with a model of individual human mobility as proposed by Yan et al.^37^ where human mobility is described, both at the individual and population level, with transition probabilities between localities governed by both a gravity-like part and a memory part. Yan et al.^37^ have shown that, in the long-time regime, the mobility patterns produced by their model are stable and robust, their model is therefore capable of simulating the disease transmission dynamics in a long duration of time.

Our approach can provide a geo-temporal spreading pattern of the epidemics that cannot be captured by a traditional homogenous epidemic model. The model predicts that the disease has a tendency to jump from (high populated) urban to urban areas before spreading to more rural areas and then subsequently spread to all neighboring locations (Fig. 1); a finding supported by other modelling studies^12,41^. In addition, this heterogeneity level of the epidemic spreading can be captured well by the time of the first arrival of the infection ($$T_{fi} )$$ (Fig. 4c, d) which is the time when the first infection is found in each location after the beginning of epidemics introduced at an origin point (the most populated location). This indicator was also used in previous work^33,42^ for comparing the epidemic outcomes between countries. Since the distribution of $$T_{fi}$$ in each location is heterogeneous, the radial-averaged of time of the first arrival of the infection ($$\left\langle {T_{r} } \right\rangle$$) is used to represent how disease propagates through the landscape (Fig. 2). At the early phase of epidemics, the disease spreads slowly to reach neighbor locations due to a small number of infectious individuals, but at a certain time, it spreads rapidly and widely within few days to reach distant locations.

In order to gain a better understanding of how human mobility shapes the disease spreading, we used the “relative attractiveness (RA)” based on only the transition matrix between localities to characterize the landscape of human mobility. We found that the RA map highlights highly attractive areas versus the others, the locations with a higher (lower) RA are more (less) attractive and thus having a higher (lower) tendency to be visited by infectious individuals (Fig. 4a, b). In addition, the $T_{fi}$ map can provide information on how disease spreads in both temporal and spatial scales. By integrating the information from the RA and $T_{fi}$ maps, our analysis indicates that the disease is more likely to reach the highly attractive areas faster than the low attractive areas. Interestingly, the relation between RA and $T_{fi}$ follows the truncated power equation. The fitting parameter $t_{1}$ can also be used to illustrate the time of the first arrival of the infection spreading into ‘flat lands’, defined as locations with RA = 1. At the individual level, we tracked the movement trajectories of infectious individuals and recorded the visitation frequency of infectious individuals in each location (Figure S5). We found that the number of times that infectious individuals visit a location is proportional to the value of RA of that location. The locations with higher RA are more likely to be visited by infectious individuals than those with a lower RA.

Our model also allows us to investigate intervention strategies at the individual level. We showed that the travel restriction of symptomatic infectious individuals provides modest results; it can decrease the epidemic size and delay the epidemic peak date (Fig. 5). The simulated incidence profile clearly shows a slowdown in the growth of the number of new infections when the travel restriction percentage increases. Our model predicts a 1–2 weeks delay of the epidemic peak when the travel restriction measure is implemented. These results are similar to those of a previous study, which examined the effect of both border and internal travel restrictions on an influenza pandemic in the United States^22^. In addition, we found that the individual waiting time also affects the epidemic dynamics (Figures S11–S13). A longer averaged waiting time of human mobility can delay the time of the first arrival of the infection in each location, extend the epidemic peak, and reduce the epidemic size.

The epidemic model presented here also has some limitations. Some parameters in the model, such as the waiting time exponent and the cutoff time in the waiting time distribution, and the memory parameter, are very difficult to measure and therefore need to be estimated. The values of these parameters depend on human mobility behavior and hence can vary in different countries^43^. These may make it difficult to apply the proposed model in a different setting. However, in order to investigate how the individual human mobility spatio-temporally shapes the disease transmission dynamics in different geographical structures, in this study, these human mobility parameters were kept the same when simulating the disease transmission in both Belgium and Martinique. Besides, since our model keeps track of each individual in the population, it can be time-consuming and expensive when simulating the model in a country with a very large population. However, we have performed a sensitivity analysis for the change in the population density (Figures S8–S10), we found that when the population size increases, the epidemic peaks are relatively shifted to the right. This might be due to the fact that when the population size increases, there will be more susceptible individuals available in the system; it, therefore, takes longer for the epidemic to reach its peak. However, the percentages of the number of infected individuals are not much different when the population size increases, especially in Martinique. Hence, these findings could help us investigate the roles of human mobility at the country-level solely by scaling down the population size in the simulations. For the sake of simplicity, we also neglected the human migration between countries. Furthermore, the model does not consider the heterogeneity in the population, such as ages, households, and social contacts; as well as specific locations of, for example, schools, homes, or workplaces. All of these factors may affect disease transmission^4,6,44–46^. Finally, we did not consider a change in human behavior under the threat of the epidemic; for instance, in reality, during an epidemic, people may avoid travelling to crowded locations.

Due to the explicit representation of human individuals, our model can allow us to understand the mechanism hidden in human mobility that affects the epidemics both at population and individual levels. Future work could employ the model to investigate the characteristics of the areas or the behaviors of an infectious individual which accelerates disease transmission or a super-spreader. Moreover, it can be used to explore other intervention strategies for containing or mitigating epidemics such as quarantine of infectious individuals and targeted vaccination. We believe that the proposed model would be beneficial for the modeling of disease spreading and imperative for the preparedness plans.

## Methods

### Population data

The spread of infection was studied in two contrasted areas, Belgium and Martinique, to investigate different human mobility landscapes. Belgium, a European country, covers an area of 30,688 km^2^ with a population of 11,250,658 inhabitants, and Martinique is a French territory of 1,128 km^2^ with 408,596 inhabitants. The geographical areas of Belgium and Martinique were divided into 2,252 and 86 grids with the 2.5-arcminute resolution (5 km at the equator), respectively. The grid structures and population sizes within each grid cell (locality) for both Belgium and Martinique were obtained from the socioeconomic data and applications center (SEDAC)^43^.

### The SEIR epidemic model with human mobility

**Figure 6:**
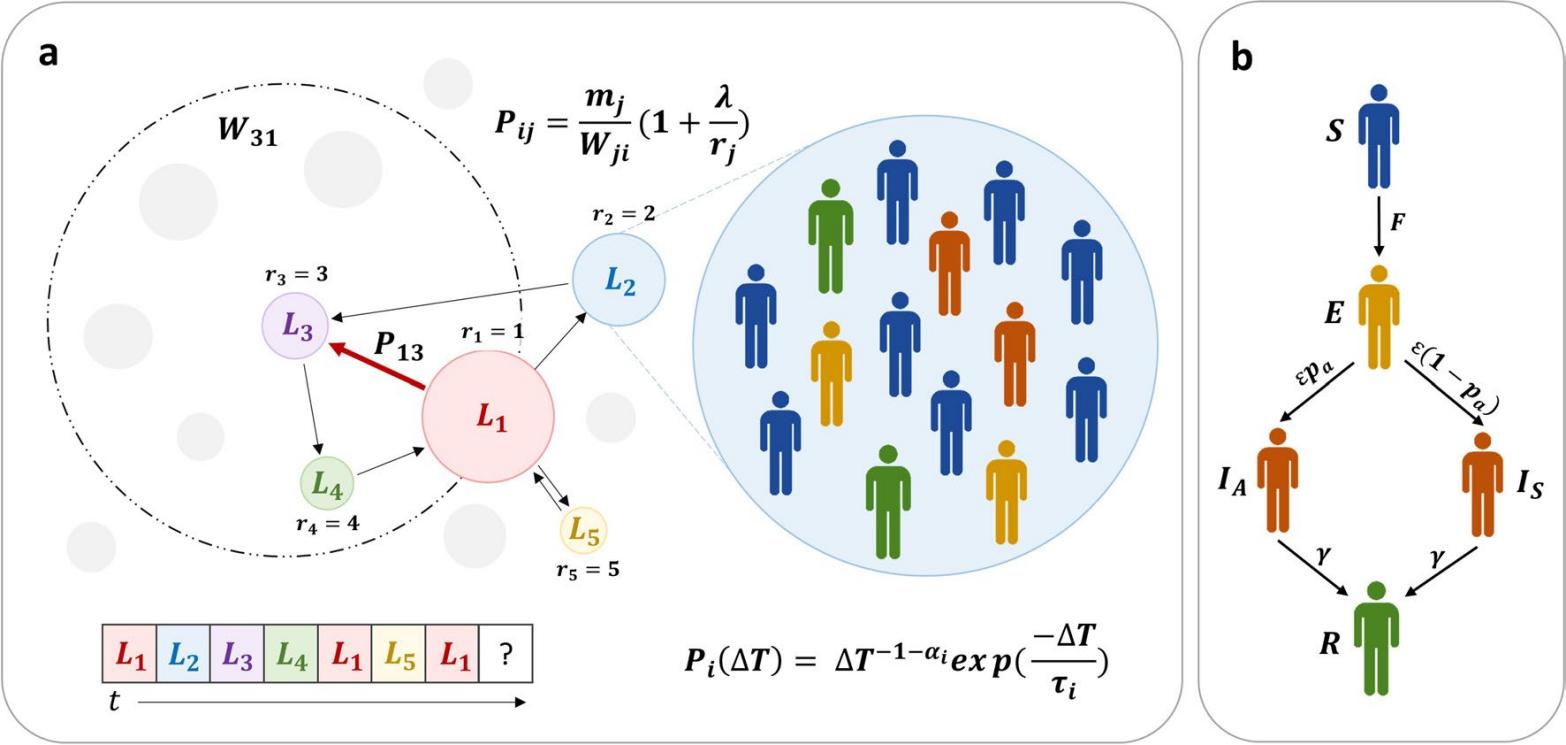
Schematic illustration of the epidemic model incorporating individual human mobility. (**a**) Five visited locations, for example, are denoted by letters $L_{1} - L_{5}$ with different colors. The population density of a location is proportional to the size of the circle that contains the letter. An example of a sequence of letters indicating the individual’s movement trajectory, $L_{1} \to L_{2} \to L_{3} \to L_{4} \to L_{5} \to L_{1} \to \cdots$ , is shown at the bottom. The transition probability for an individual moving from location $i$ to location $j$ is governed by two elements, namely, a gravity-like part ($B_{ij} = m_{j} /W_{ji}$) and a memory effect $\left( {A_{j} = 1 +\uplambda {/}r_{j} } \right)$. If location $j$ has never been visited before (grey circle), its $A_{j}$ value is assumed to be unity, $A_{j} = 1$; thus, it will be visited with $p_{ij} = m_{j} /W_{ji}$. For the previously visited location *j*, it can be visited again with $p_{ij} = \frac{{m_{j} }}{{W_{ji} }}\left( {1 + \frac{\uplambda }{{r_{j} }}} \right)$, where $r_{j}$ is an index indicating that location $j$ is the *r*th visited location. At a certain time, each location contains a number of human individuals who will stay at the location for different periods of time drawn from the waiting time distribution ${\text{P}}\left( {\Delta T} \right)$^23^. The figure was adapted from the model proposed by Yan et al.^37^. (**b**) Based on the individual’s disease transmission dynamics, individuals are categorized into five compartments, namely, the susceptible ($S$), exposed ($E$), asymptomatic infectious ($I_{A}$), symptomatic infectious ($I_{s}$), and recovered ($R$) compartments. Individuals in the exposed compartment move to the asymptomatic infectious compartment with the probability $p_{a}$.

To investigate the roles of human mobility on the spreading of epidemics, we consider the spread of human influenza over an area comprising of $N$ localities or locations, each of which with a population $m_{i}$ such that the total population of the area is, $M = \mathop \sum \nolimits_{i = 1}^{N} m_{i}$. Two processes are considered: (1) individual human mobility between distinct localities using the model adapted from the model proposed by Yan et al.^37^ (Fig. 6a) and (2) within each locality, the transmission of infection between individuals is described using a compartmental SEIR model (Fig. 6b).

#### Human mobility

The locations in the study area are represented in Fig. 6a by circles of size proportional to their population size $m_{i}$. In this model, the probability that an individual initially at location $i$ at time $t$ travels to location $j$ at time $t + \Delta T$ is given by ${\text{G}}_{ji} \left( {t,t + \Delta T} \right) = {\text{P}}_{i} \left( {\Delta T} \right) \times p_{ij}$, where ${\text{P}}_{i} \left( {\Delta T} \right)$ is the distribution of waiting times at the initial location before moving and $p_{ij}$ is the transition probability. The distribution of waiting times is given by^23^,

1 $$\begin{array}{*{20}c} {{\text{P}}_{i} \left( {\Delta T} \right) = \Delta {\text{T}}^{{ - 1 - {\upalpha }_{i} }} \exp \left( { - \frac{{\Delta {\text{T}}}}{{\tau_{i} }}} \right),} \\ \end{array}$$

where ${\upalpha }_{i}$ is the waiting time exponent and $\tau_{i}$ is the cutoff time of the location $i$. For simplicity, we assumed in all simulations that ${\text{P}}_{i} \left( {\Delta T} \right)$ is independent of the location and, therefore, identical for all locations (${\text{P}}_{i} (\Delta T) = {\text{P}}(\Delta T)$). Details of the method for estimating the waiting time parameters, ${\upalpha }$ and $\tau$, are provided in the supplement information.

The transition probability for traveling from location *i* to location *j* is governed by two elements. The first element is a gravity-like contribution,

2 $$\begin{array}{*{20}c} {B_{ij} = \frac{{m_{j} }}{{W_{ji} }},} \\ \end{array}$$

where $m_{j}$ is the population of destination location $j$ and $W_{ji}$ is the total population within the circular region centered at location $j$ having the radius equal to the distance between locations *i* and *j* as illustrated by the dashed circle in Fig. 6a. In this model, the transition matrix $B_{ij}$ is characterized by peaks of the points more likely to be visited at short and long distances from the origin (jump transitions) than it would be in the case of the classical gravity-like model. This heterogeneity of the transition matrix would be more marked when the distribution of population densities is heterogeneous. The second part is a memory effect

3 $$\begin{array}{*{20}c} {A_{j} = 1 + \frac{{\uplambda }}{{r_{j} }},} \\ \end{array}$$

where $r_{j}$ is the index indicating the order *r*th of the visit of location $j$ along the movement trajectory (shown at the bottom of Fig. 6a), with $r = 1$ for the home location, and λ is a constant parameter representing the strength of the memory effect. Therefore, the transition probability that an individual travels from location $i$ to locations $j$ is,

4 $$\begin{array}{*{20}c} {p_{ij} = \frac{{m_{j} }}{{W_{ji} }}\left( {1 + \frac{\uplambda }{{r_{j} }}} \right).} \\ \end{array}$$

Note that $A_{j} = 1$ when location $j$ has never been visited (gray circle) before. It follows that the probability for an individual exploring new locations is simply, $p_{ij} = m_{j} /W_{ji}$. Full description of the model parameters and their meaning can be found in^37^.

#### SEIR individual-based model

The population in each location (enlarged circle in Fig. 6a) is classified into four epidemiological classes: susceptible (*S*), exposed (*E*), infectious (subdivided into asymptomatic (*I*_*A*_) and symptomatic infectious (*I*_*S*_)), and recovered (*R*) (Fig. 6b) ^4^. Within each location, susceptible individuals become infected, and progress to the exposed class, following contacts with a symptomatic or an asymptomatic infectious individual staying at the same location as described by the force of infection $F_{i}$ given by

5 $$\begin{array}{*{20}c} {F_{i} = \frac{{\beta I_{S,i} + \omega \beta I_{A,i} }}{{m_{i} }},} \\ \end{array}$$

where $m_{i}$ is the population size at location *i*, $\beta$ is the disease transmission rate, and $\omega$ is a scaling parameter that takes into account the reduced infectiousness of asymptomatic infectious individuals. It is worth noting that, since influenza viruses spread from person to person primarily through large-particle respiratory droplet transmission, which requires close contact between source and recipient persons. In our study, we, therefore, assumed that the influenza transmission is the frequency-dependent transmission. Such an assumption was also used to predict the spread of influenza in several studies^6,20,38,41,47,48^. After being infected, exposed individuals become either asymptomatic (with a probability *p*_*a*_) or symptomatic infectious (with a probability 1 − *p*_*a*_) at a rate $\varepsilon$, which is inversely proportional to the incubation period. Both symptomatic and asymptomatic infectious individuals recover from the disease at a rate, *γ,* which is inversely proportional to the infectious period, and have lifelong immunity. The disease transmission rate was calculated using the following relationship^41^: $R_{0} = \frac{\beta }{\gamma }\left[ {\left( {1 - p_{a} } \right) + p_{a} \omega } \right]$.

The descriptions and values of all parameters used in the model are summarized in Table 1.

**Table 1:** A summary of the parameters and their default values used in the model.

Definition	Symbol	Values	Refs
Basic reproduction number	$R_{0}$	1.9	^41^
Disease transmission rate	$\beta$	0.7585 day^−1^	^41^
Average latency period	$\varepsilon^{ - 1}$	2 days	^41^
Average infectious period	$\gamma^{ - 1}$	3 days	^20,41^
Probability of asymptomatic disease	$\rho_{a}$	33%	^20,41^
Reduced transmission for asymptomatic infectious individuals	$\omega$	50%	^20,41^
Memory parameter	$\uplambda$	25	^37^
Waiting time exponent	$\alpha$	0.4461	*
Cutoff time	$\tau$	10^4^ h ($\sim$ 1.14 years)	*
Average waiting time	$\left\langle {\Delta T} \right\rangle$	5 days	–
Travel restrictions of symptomatic infectious	$\eta$	0–100%	–

*See supplementary information for the estimation details.

### Mobility landscape: the relative attractiveness of locations (RA)

To characterize the human mobility landscape, we calculated the relative attractiveness (RA) of each location of the study area. To this end, we consider the Master equation describing the movements of individuals in the study area as,

6 $$\begin{array}{*{20}c} {\frac{{d\overset{\lower0.5em\hbox{$\smash{\scriptscriptstyle\rightharpoonup}$}} {P} }}{dt} = \left[ {{\varvec{B}}^{{\varvec{t}}} - {\varvec{K}}} \right]\overset{\lower0.5em\hbox{$\smash{\scriptscriptstyle\rightharpoonup}$}} {P} ,} \\ \end{array}$$

where $$\overset{\lower0.5em\hbox{$\smash{\scriptscriptstyle\rightharpoonup}$}} {P}$$ is a column vector of probabilities of finding individuals at location $$i$$ and time $$t$$, $${\varvec{B}}^{{\varvec{t}}}$$ is the transpose of the matrix $${\varvec{B}}$$ **(**of the gravity-like part of the mobility) with elements $$B_{ij} = m_{j} /W_{ji} { }$$ and the matrix **K** is given as,

7 $$K_{ij} = \left\{ {\begin{array}{*{20}l} {\mathop \sum \limits_{j = 1}^{N} B_{ij} {,}} \hfill & {{\text{for}}\, i = j} \hfill \\ {0,} \hfill & {{\text{for}}\, i \ne j} \hfill \\ \end{array} } \right.$$

where *N* is the total number of locations. The matrix $${\varvec{B}}^{{\varvec{t}}} - {\varvec{K}}$$ describes the fluxes of individuals traveling forward and backward between all locations. At the stationary state, the time variation of the probability vector $$\overset{\lower0.5em\hbox{$\smash{\scriptscriptstyle\rightharpoonup}$}} {P}$$ is zero, i.e., $$\left[ {{\varvec{B}}^{{\varvec{t}}} - {\varvec{K}}} \right]\overset{\lower0.5em\hbox{$\smash{\scriptscriptstyle\rightharpoonup}$}} {P} = 0$$. It thus follows that the stationary probability is obtained as, $$\overset{\lower0.5em\hbox{$\smash{\scriptscriptstyle\rightharpoonup}$}} {P}_{st} = \overset{\lower0.5em\hbox{$\smash{\scriptscriptstyle\rightharpoonup}$}} {U}$$, where $$\overset{\lower0.5em\hbox{$\smash{\scriptscriptstyle\rightharpoonup}$}} {U}$$ is the (normalized) eigenvector of $${\varvec{B}}^{{\varvec{t}}} - {\varvec{K}}$$ associated to the zero eigenvalue. Finally, the relative attractiveness $$RA_{i}$$ of location $$i$$ can be defined as a ratio of the stationary probability of finding individuals at location $$i$$, $$P_{st,i}$$ , and the equi-probability, $$1/N,$$ of finding individuals at any location;

8 $$\begin{array}{*{20}c} {RA_{i} = \frac{{P_{st,i} }}{{1{/}N}} = U_{i} \times N.} \\ \end{array}$$

Therefore, locations with $RA_{i} > 1$ have a higher propensity to be visited by individuals than those with $RA_{i} < 1.$

### Time of the first arrival of the infection

When it comes to an epidemic, we are mainly interested in knowing how the infection or the transmission chain would spread and spread with what associated speeds. A commonly used approach to analyze an epidemic spread in terms of directions and velocities is the “trend surface analysis (TSA)”^29–33^, to name just one. Unfortunately, such a method cannot be used to predict the profile of the spread and it becomes inappropriate for analyzing the spread of an epidemic due to the heterogeneity of host movements. To address how epidemics spread through different locations, we used the time of the first arrival of the infection, $T_{fi} {(}i {|} j)$, which is the average elapsed time that an infection started out at location $j$ reaches location $i$ for the first time. In all simulations reported in this work, we assumed that the first infected location $j$ was the most populated one so as to use $T_{fi} \left( i \right)$ instead of $T_{fi} {(}i {|} j)$, for simplicity. Due to different pathways of infection between two locations, the distribution of velocity is heterogeneous. To have an idea of how the expansion of epidemics is, we computed the radial-averaged time of the first arrival of the infection $\left\langle {T_{r} } \right\rangle$ for all locations at radius *r* from the starting point of epidemics. Then the radial speed of expansion $v\left( r \right)$ was calculated by

9 $$\begin{array}{*{20}c} {\frac{1}{v(r)} = \frac{{\partial \left\langle {T_{r} } \right\rangle }}{\partial r}.} \\ \end{array}$$

### Mean square displacement

In the simulations, we tracked the movement trajectories of infectious individuals to understand how infectious individuals move after they got the infection. We recorded the sequences of visited locations, step lengths, and times that infectious individuals spent at each location. To analyze the mobility of infectious individuals, we calculated the time-averaged mean square displacement (MSD), $\delta^{2} \left( \Delta \right)$, of each infectious individual^49^;

10 $$\begin{array}{*{20}c} {\delta_{i}^{2} (\Delta ) = \frac{1}{{T_{i} - \Delta }}\mathop \sum \limits_{t = 0}^{{T_{i} - \Delta }} \left( {r_{i} \left( {t + \Delta } \right) - r_{i} \left( t \right)} \right)^{2} ,} \\ \end{array}$$

where $\delta_{i}^{2} \left( \Delta \right)$ is MSD of infectious individual $i$ at a different lag time (Δ), $T_{i}$ is a tracking period, a time duration since individual $i$ got infection to the time when he/she recovered, Δ is a lag time ($\Delta < T_{i}$), $r_{i} \left( t \right)$ is a spatial displacement of infectious individual $i$ at time $t$ after being infectious. We then averaged the MSD of all infectious individuals, $\left\langle {\delta^{2} (\Delta )} \right\rangle$. This MSD describes how far, on average, infectious individuals travel during their infectious duration.

### Simulation details

In the simulation, individuals in each location initially stay in particular locations for certain periods of time drawn from the truncated power law (Eq. 1) and then move to other locations following the transition probability (Eq. 4). All visited locations and their orders of the visit of each individual are recorded for calculating the memory effect.

Before introducing an infection into the model simulations, human mobility was first pre-equilibrated by simulating the human mobility model until the total human mobility flux of each location reaches a constant, i.e., the number of individuals moving in equals to the number of individuals moving out. Unless stated otherwise, we assumed that the first infectious individual is initially introduced in the most densely populated location. At each time step of one hour, the disease transmission dynamics within each location was simulated using the tau-leaping method^50–52^. Besides spreading within a location, the disease can be transmitted spatially to other locations via human mobility in which its speed relies on the waiting time distributions and the transition probability (Eqs. 1, 4). In this model, we assumed that the dynamics of human birth and death are much slower than the dynamics of the epidemics and human mobility, and there is no emigration and no immigration; thus, the human population size is constant. The descriptions and values of all parameters used in the model are summarized in Table 1. All simulations were performed using MATLAB software (version R2018b, The MathWorks, Inc) running on personal computers. All maps showing the spatio-temporal disease spreading patterns in Belgium and Martinique were also generated with the use of MATLAB software (version R2018b, The MathWorks, Inc).

In this study, we also examined the effects of restriction on symptomatic infectious individual movement in Belgium with $\left\langle {\Delta T} \right\rangle = 5$ days. The percentage of symptomatic infectious individuals restricted to travel to other locations, $\eta$, was varied from 0%, 20%, 50%, 70%, and 100%. Because infectious individuals partly become asymptomatic with the probability $\rho_{a}$, therefore, the proportion of travelling infectious individuals, $\theta$, is

11 $$\begin{array}{*{20}c} {\theta = \rho_{a} + (1 - \rho_{a} )(1 - \eta ).} \\ \end{array}$$

This means that when all symptomatic infectious individuals are banned from traveling ($\eta = 100\%$), there will be only the mobility of asymptomatic infectious individuals ($\theta$ = $\rho_{a}$). On the other hands, when $\eta = 0\% ,$ everyone moves normally ($\theta$ = 1).

## Supplementary information

Supplementary file1

## Data Availability

The authors declare that the data supporting the findings of this study are available within the paper and its Supplementary Information file. Computer code are available from the authors upon reasonable request.

## References

[1] Jones KE (2008). Global trends in emerging infectious diseases. Nature.

[2] Bloom DE, Cadarette D (2019). Infectious disease threats in the 21st century: strengthening the global response. Front. Immunol..

[3] Altizer S (2006). Seasonality and the dynamics of infectious diseases. Ecol. Lett..

[4] Mossong J (2008). Social contacts and mixing patterns relevant to the spread of infectious diseases. PLoS Med..

[5] Dushoff J, Levin S (1995). The effects of population heterogeneity on disease invasion. Math. Biosci..

[6] Merler S, Ajelli M (2009). The role of population heterogeneity and human mobility in the spread of pandemic influenza. Proc. R. Soc. B Biol. Sci..

[7] Yang Z, Cui A-X, Zhou T (2011). Impact of heterogeneous human activities on epidemic spreading. Phys. A Stat. Mech. Appl..

[8] Meloni S (2011). Modeling human mobility responses to the large-scale spreading of infectious diseases. Sci. Rep..

[9] Balcan D (2010). Modeling the spatial spread of infectious diseases: the global epidemic and mobility computational model. J. Comput. Sci..

[10] Charu V (2017). Human mobility and the spatial transmission of influenza in the United States. PLoS Comput. Biol..

[11] Colizza V, Vespignani A (2008). Epidemic modeling in metapopulation systems with heterogeneous coupling pattern: theory and simulations. J. Theor. Biol..

[12] Zhang N (2018). A human behavior integrated hierarchical model of airborne disease transmission in a large city. Build. Environ..

[13] Balcan D (2009). Multiscale mobility networks and the spatial spreading of infectious diseases. Proc. Natl. Acad. Sci..

[14] Funk S, Salathé M, Jansen VA (2010). Modelling the influence of human behaviour on the spread of infectious diseases: a review. J. R. Soc. Interface.

[15] Ni S, Weng W (2009). Impact of travel patterns on epidemic dynamics in heterogeneous spatial metapopulation networks. Phys. Rev. E.

[16] Tang M, Liu Z, Li B (2009). Epidemic spreading by objective traveling. EPL (Europhys. Lett.).

[17] Dalziel BD, Pourbohloul B, Ellner SP (2013). Human mobility patterns predict divergent epidemic dynamics among cities. Proc. R. Soc. B Biol. Sci..

[18] Wang B (2012). Safety-information-driven human mobility patterns with metapopulation epidemic dynamics. Sci. Rep..

[19] Epstein JM (2007). Controlling pandemic flu: the value of international air travel restrictions. PLoS ONE.

[20] Colizza V (2007). Modeling the worldwide spread of pandemic influenza: baseline case and containment interventions. PLoS Med..

[21] Bajardi P (2011). Human mobility networks, travel restrictions, and the global spread of 2009 H1N1 pandemic. PLoS ONE.

[22] Ferguson NM (2006). Strategies for mitigating an influenza pandemic. Nature.

[23] Gonzalez MC, Hidalgo CA, Barabasi A-L (2008). Understanding individual human mobility patterns. Nature.

[24] Brockmann D, Hufnagel L, Geisel T (2006). The scaling laws of human travel. Nature.

[25] Song C (2010). Modelling the scaling properties of human mobility. Nat. Phys..

[26] Szell M (2012). Understanding mobility in a social petri dish. Sci. Rep..

[27] Schneider CM (2013). Unravelling daily human mobility motifs. J. R. Soc. Interface.

[28] Gallotti R (2016). A stochastic model of randomly accelerated walkers for human mobility. Nat. Commun..

[29] Jung W-S, Wang F, Stanley HE (2008). Gravity model in the Korean highway. EPL (Europhys. Lett.).

[30] Barbosa H (2015). The effect of recency to human mobility. EPJ Data Sci..

[31] Pappalardo L, Rinzivillo S, Simini F (2016). Human mobility modelling: exploration and preferential return meet the gravity model. Procedia Comput. Sci..

[32] Pappalardo L (2015). Returners and explorers dichotomy in human mobility. Nat. Commun..

[33] Tizzoni M (2014). On the use of human mobility proxies for modeling epidemics. PLoS Comput. Biol..

[34] Wang L (2013). How human location-specific contact patterns impact spatial transmission between populations?. Sci. Rep..

[35] Poletto C, Tizzoni M, Colizza V (2013). Human mobility and time spent at destination: impact on spatial epidemic spreading. J. Theor. Biol..

[36] Zhang C (2015). Optimizing hybrid spreading in metapopulations. Sci. Rep..

[37] Yan X-Y (2017). Universal model of individual and population mobility on diverse spatial scales. Nat. Commun..

[38] Pei S (2018). Forecasting the spatial transmission of influenza in the United States. Proc. Natl. Acad. Sci..

[39] Li R, Wang W, Di Z (2017). Effects of human dynamics on epidemic spreading in Côte d’Ivoire. Phys. A Stat. Mech. Appl..

[40] Colizza V (2006). The role of the airline transportation network in the prediction and predictability of global epidemics. Proc. Natl. Acad. Sci..

[41] Ajelli M (2010). Comparing large-scale computational approaches to epidemic modeling: agent-based versus structured metapopulation models. BMC Infect. Dis..

[42] Colizza V (2006). The modeling of global epidemics: stochastic dynamics and predictability. Bull. Math. Biol..

[43] CIESIN, Gridded population of the world, version 3 (GPWv3): population count grid (NASA Socioeconomic Data and Applications Center (SEDAC), Palisades, 2005). 10.7927/H4639MPP. Accessed 1 Aug 2018.

[44] Goeyvaerts N (1893). (2018) Household members do not contact each other at random: implications for infectious disease modelling. Proc. R. Soc. B.

[45] Fraser C (2007). Estimating individual and household reproduction numbers in an emerging epidemic. PLoS ONE.

[46] Gemmetto V, Barrat A, Cattuto C (2014). Mitigation of infectious disease at school: targeted class closure vs school closure. BMC infect. Dis..

[47] Cooper BS (2006). Delaying the international spread of pandemic influenza. PLoS Med..

[48] Ferguson NM (2005). Strategies for containing an emerging influenza pandemic in Southeast Asia. Nature.

[49] Jeon J-H (2013). Anomalous diffusion and power-law relaxation of the time averaged mean squared displacement in worm-like micellar solutions. New J. Phys..

[50] Gillespie DT (2001). Approximate accelerated stochastic simulation of chemically reacting systems. J. Chem. Phys..

[51] Gillespie DT (1977). Exact stochastic simulation of coupled chemical reactions. J. Phys. Chem..

[52] Gillespie DT (1976). A general method for numerically simulating the stochastic time evolution of coupled chemical reactions. J. Comput. Phys..

