# Supplementary material for: How the individual human mobility spatio-temporally shapes the disease transmission dynamics
Source: Sci Rep. 2020 Jul 9;10:11325. doi: 10.1038/s41598-020-68230-9 (PMC7347872; doi:10.1038/s41598-020-68230-9)
Supplement: Supplementary file 1 — Supplementary file1 [file 41598_2020_68230_MOESM1_ESM.pdf]

Supplementary Information:

How the individual human mobility spatio-temporally shapes the disease transmission dynamics

Suttikiat Changruenngam[1,2], Dominique J. Bicout[2,3,*] and Charin Modchang[1,4,5,*]

[1] Biophysics Group, Department of Physics, Faculty of Science, Mahidol University, Bangkok 10400, Thailand

[2] Institut Laue-Langevin, 71 Avenue des Martyrs, 38042, Grenoble, France

[3] EPSP, TIMC Laboratory, UMR CNRS 5525 Grenoble Alpes University, VetAgro Sup, Grenoble, France

[4]Centre of Excellence in Mathematics, CHE, Bangkok 10400, Thailand

[5]Thailand Center of Excellence in Physics, CHE, 328 Si Ayutthaya Road, Bangkok 10400, Thailand

* To whom correspondence should be addressed. Email: charin.mod@mahidol.edu (CM), bicout@ill.fr (DJB)

Comparison with the homogeneous epidemic model

To investigate the roles of human mobility in the disease transmission dynamics, we compared the time course of the number of infectious and of recovered individuals in Belgium simulated stochastically using the homogeneous compartmental SEIR model and the epidemic model that incorporates human mobility (**Figure S1**). The dynamics of the disease transmission in the homogeneous SEIR model are governed by the following set of ordinary differential equations.

$$\frac{dS}{dt} = -(\beta I_S + r\beta I_A)\frac{S}{N},$$

$$\frac{dE}{dt} = (\beta I_S + \omega\beta I_A)\frac{S}{N} - \varepsilon E,$$

$$\frac{dI_S}{dt} = (1 - p_a)\varepsilon E - \gamma I_S, \qquad \text{(S1)}$$

$$\frac{dI_A}{dt} = p_a \varepsilon E - \gamma I_A,$$

$$\frac{dR}{dt} = \gamma(I_S + I_A),$$

where S, E, I_S, I_A, R are the number of susceptible, exposed, symptomatic infectious, asymptomatic infectious, and recovered individuals, respectively. β is a disease transmission rate, and ω is a scaling parameter taking into account the reduced infectiousness of asymptomatic infectious individuals. p_a is the probability of an exposed individual to become an asymptomatic infectious individual. ε is an incubation rate, which is inversely proportional to the incubation period. γ is a recovery rate, which is inversely proportional to the infectious period, and N is the total population size, $N = S + E + I_S + I_A + R$.

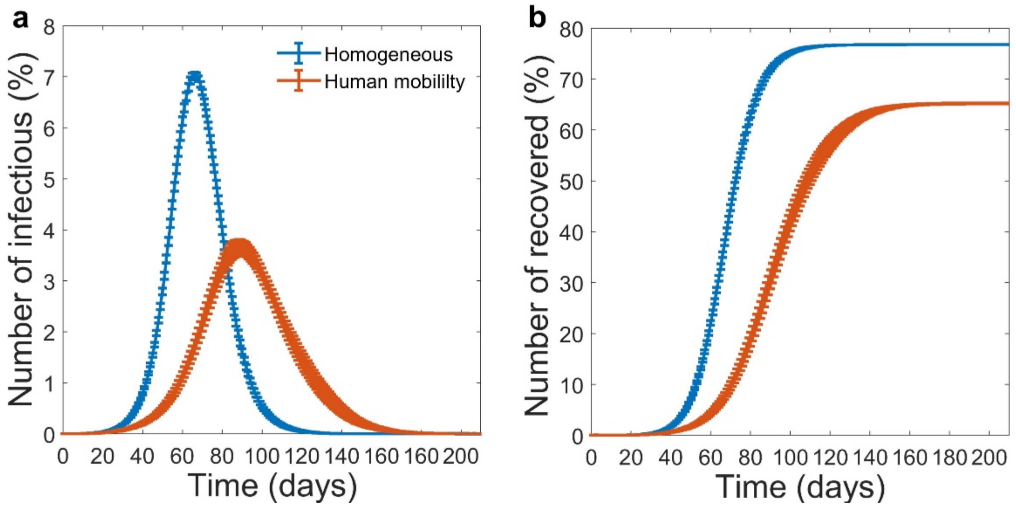

Figure S1. Comparison of the epidemic curve and size. (a) The number of infectious individuals and **(b)** the number of recovered individuals simulated stochastically using the homogeneous epidemic model (blue line) and the epidemic model that takes into account human mobility (red line). The population size simulated in both models is 1.1×10^7 and the averaged waiting time of 5 days is used for the human mobility model. The errorbars indicate the standard error of the mean.

We found that the dynamics of human mobility can affect epidemic profiles. As compared to the homogeneous model, spatial heterogeneity arising due to human mobility can delay the epidemic peak of up to 15 days with a flatter and wider epidemic curve (**Figure S1a**). This slower speed of the epidemic spread is partly due to an effect of the waiting time, which retards the movement of infectious individuals. Moreover, the epidemic model that integrates human mobility predicts 8.7% less the total number of recovered individuals at the equilibrium as compared to the homogeneous model (**Figure S1b**).

Homogeneous epidemic model: Deterministic versus stochastic approaches

Generally, a homogeneous epidemic model can be simulated using either a deterministic or a stochastic approach [1]. We investigated under what conditions stochastic simulations give rise to epidemic curves that are markedly different from those obtained from deterministic simulations. To test the hypothesis, we run the homogeneous model with different numbers of initial infectious individuals. We simulated the homogeneous model deterministically by numerically solving equation S1 using the Euler method while the tau-leaping method [2-4] was used to simulate the model stochastically.

We found that the stochasticity in disease transmission is dominated when the number of infectious individuals is very low, especially at the early time of the epidemics. However, the results of the deterministic simulations will be identical to the results of the stochastic simulations when the number of initial infectious individuals is sufficiently high. As shown in **Figure S2**, for the deterministic simulations, using lower numbers of initial infectious individuals only shift the epidemic peak to the right but does not significantly affect the shape of the curve. In contrast, using lower numbers of infectious individuals in the stochastic simulations does not only shift the epidemic peak to the right but also decreases the epidemic peak and widens the epidemic curve. This is because when the number of initial infectious individuals is very low, there is a very high chance for the disease to become extinct before a sustained chain of transmission can be established. However, as expected, the results of the stochastic simulations are identical to the results of the deterministic simulations in the case when the number of initial infectious individuals is high enough (e.g., $I_{int} = 100$). This might imply that the deterministic simulations should provide the results that are identical (or at least similar) to the results of the stochastic simulations when the numbers of human individuals in all epidemiological compartments are high.

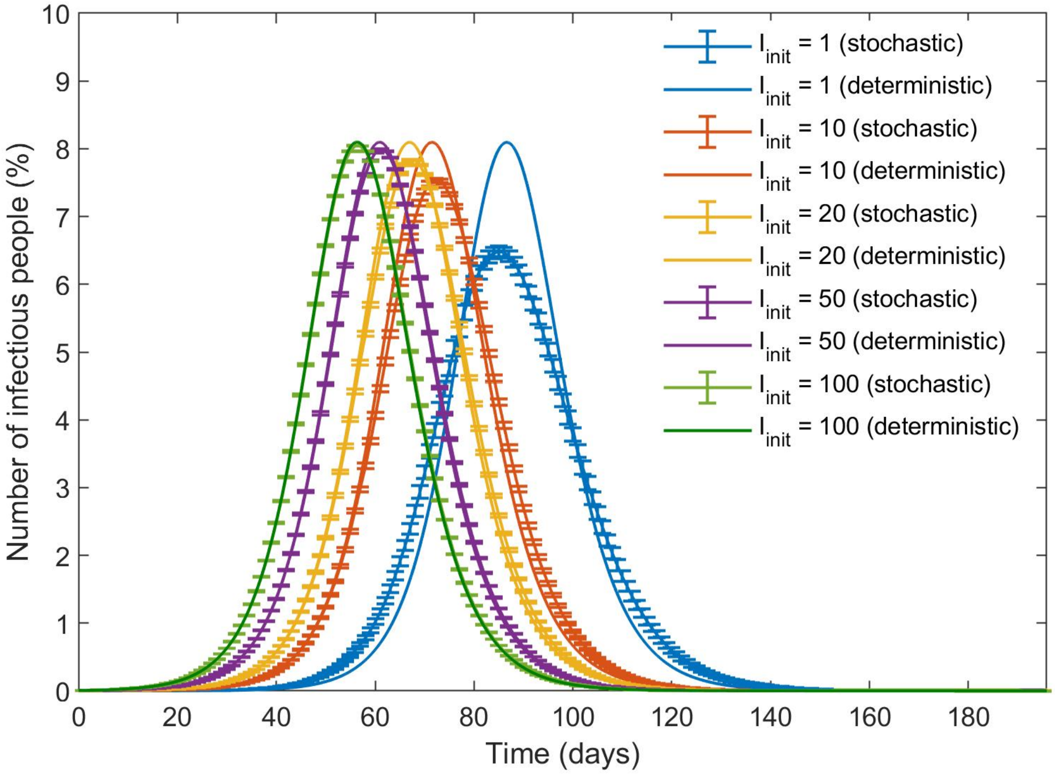

Figure S2. Epidemic curves simulated from the homogeneous epidemic model. The homogeneous model was simulated using both the deterministic and stochastic approaches with different numbers of initial infectious individuals (I_{int}) and $N = 1.1 \times 10^7$.

Estimating the waiting time parameters

In our human mobility model, we assumed that the waiting time distribution $P_i(\Delta T)$ (equation 1 in the main text) is identical for all locations i. Thus, an individual will stay at a particular location for a period of time of ΔT drawn from the following truncated power law distribution [5]

$$P(\Delta T) = \Delta T^{-1-\alpha} \exp\left(\frac{-\Delta T}{\tau}\right), \quad (S2)$$

The truncated power law distribution is governed by two parameters, namely, an exponent (α), and cutoff time (τ). The cutoff time roundly indicates the possible maximum waiting time in which an individual stays at a particular location.

Using the phone call data, where the individual human locations were estimated by the associated locations of the cell phone towers, Gonzalez et al. found that the cutoff time was 48 days [5]. However, since the cell phone tower used in the previous study served an area of only approximately 3 km^2, which is smaller than the grid size of 25 km^2 used in our study, we, therefore, rescaled the cutoff time by assuming that the cutoff time is proportional to the area of a location. In our study, we, therefore, used $\tau = 10^4$ hours. In order to estimate α, we considered an average value of the waiting time ($< \Delta T >$) given by

$$< \Delta T > = \int_1^{\infty} \Delta T^{-\alpha} \exp\left(\frac{-\Delta T}{\tau}\right) d(\Delta T). \quad (S3)$$

By numerically integrating equation S3, the numerical relationship between $< \Delta T >$ and α was obtained, as shown in **Figure S3**. We found that $< \Delta T > = 1, 3, 5, 10,$ and 30 days are associated to α = 0.7524, 0.5392, 0.4461, and 0.1059, respectively.

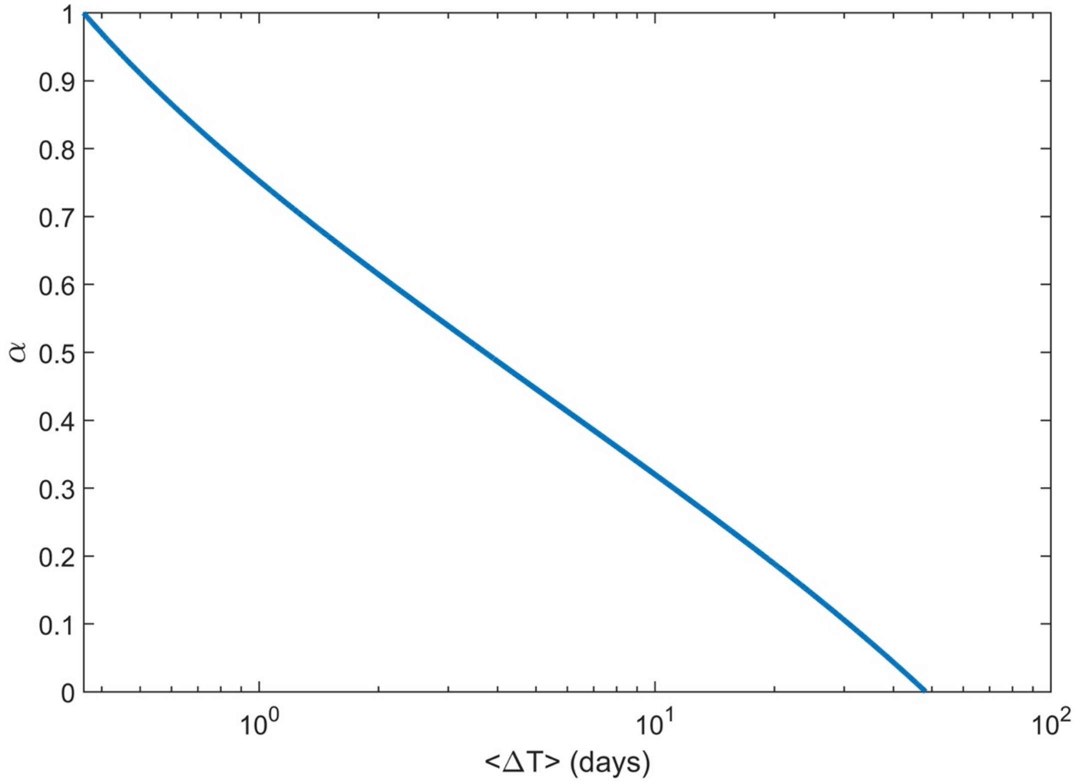

Figure S3. Estimating the waiting time parameters. The numerical relationship between α and $< \Delta T >$.

Redial speed of disease spreading

To quantify the disease spreading, we computed the radial-averaged time of the first arrival of infection ($< T_r >$) in a location at radius r centered at the first infected location and the corresponding radial speed of spread (v_r) as shown in **Figure S4** for Belgium (above) and Martinique (bellow). We also examined the effect of average waiting time, $< \Delta T >$, varied from 1 day, 3 days, 5 days, 10 days, and 30 days shown in different colors. The curves of $< T_r >$ are shifted up vertically, and the slope of v_r is higher for both Belgium and Martinique when $< \Delta T >$ increases. However, in Martinique, $< \Delta T >$ does not significantly affect the disease spreading dynamics, which might be due to the fact that the distribution of population in Martinique is quite homogeneous as compared to that in Belgium.

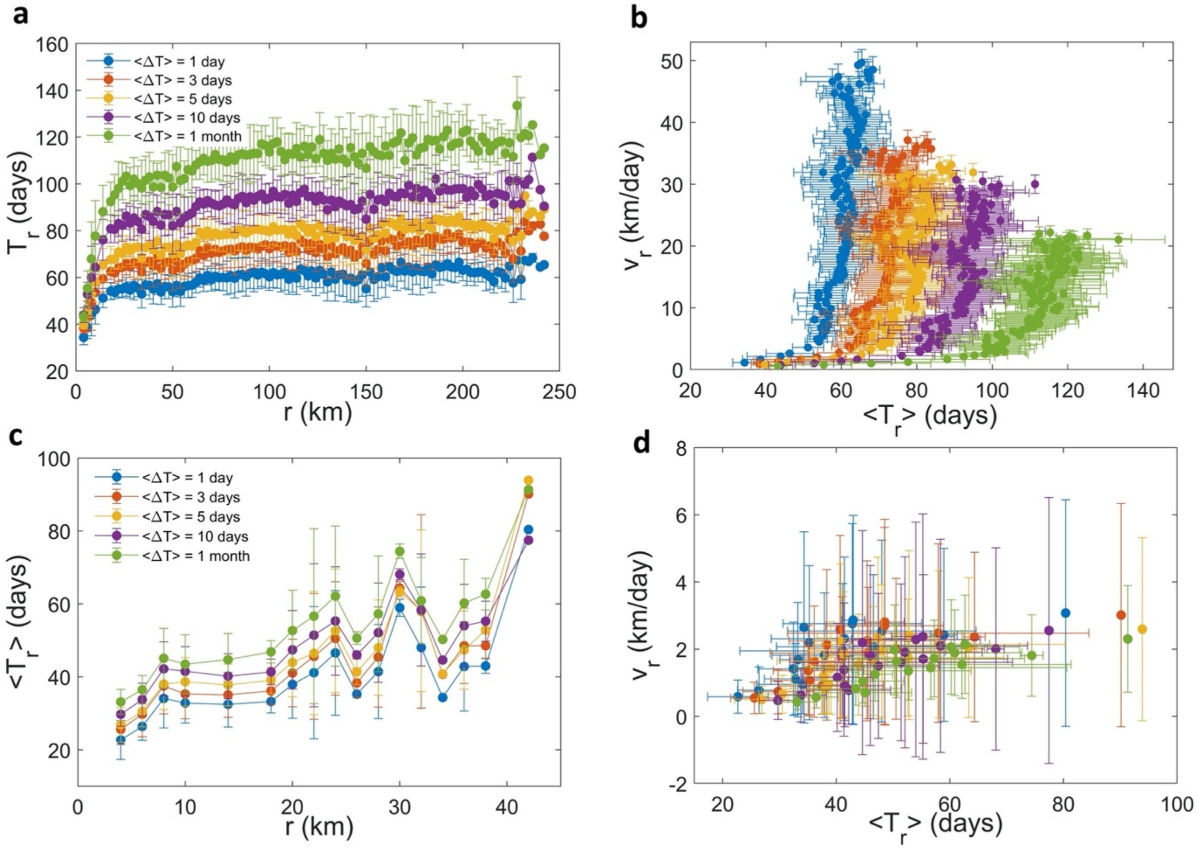

Figure S4. Radial speed of disease spread. (a, c) The radial-averaged time of the first arrival of infection in each location at radius r centered at the first infected location and **(b, d)** the corresponding radial speed of spread for Belgium (above) and Martinique (below) at different $< \Delta T >$.

Mobility of infectious individuals

To understand the mobility of infectious individuals, we measured the visitation frequency of infectious individuals by tracking their movement trajectories both in Belgium and Martinique with $< \Delta T >= 5$ days (**Figure S5**). We found that the number of times that infectious individuals visit each location is consistent with the RA map shown in **Figure 7a** and **7b** for Belgium and Martinique, respectively. The locations with larger RA are likely to be visited more frequently by infectious individuals than those with a smaller RA.

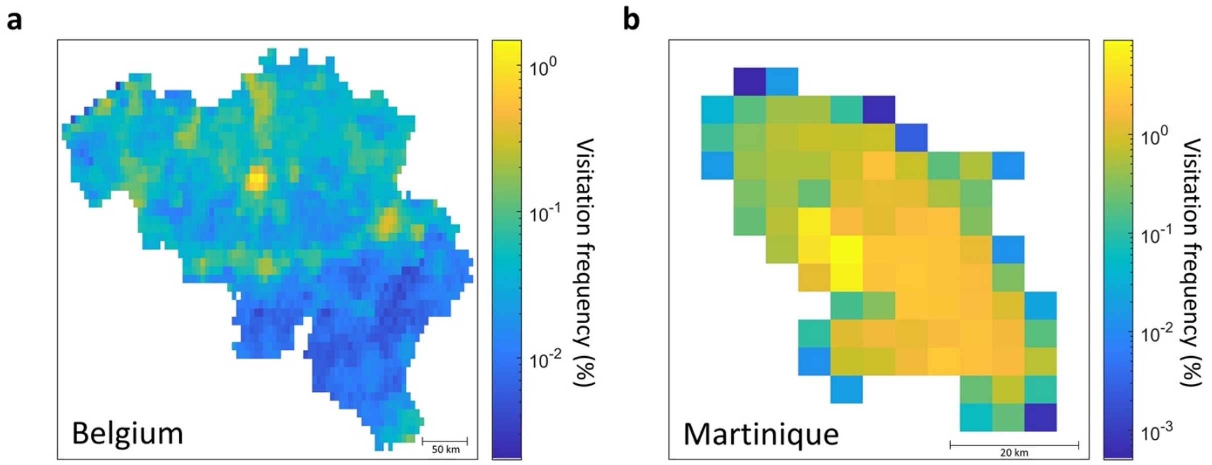

Figure S5. Visitation frequency. (a-b) The maps show the infectious-individual visitation frequency of locations in Belgium and Martinique (normalized to 100%), respectively. The visitation frequency was recorded at the end of epidemics; 5 months.

Moreover, we computed the probability distribution of the number of locations visited by an infectious individual, $P(S)$, (**Figure S6a** and **S6c**) and the probability distribution of the number of step lengths among the visited locations of an infectious individual, $P(L)$, (**Figure S6b** and **S6d**) for Belgium (above) and Martinique (below). Most infectious individuals have a tendency to travel among a few locations with a small number of step lengths, and, in contrast, few of them visit several locations with a large number of step lengths.

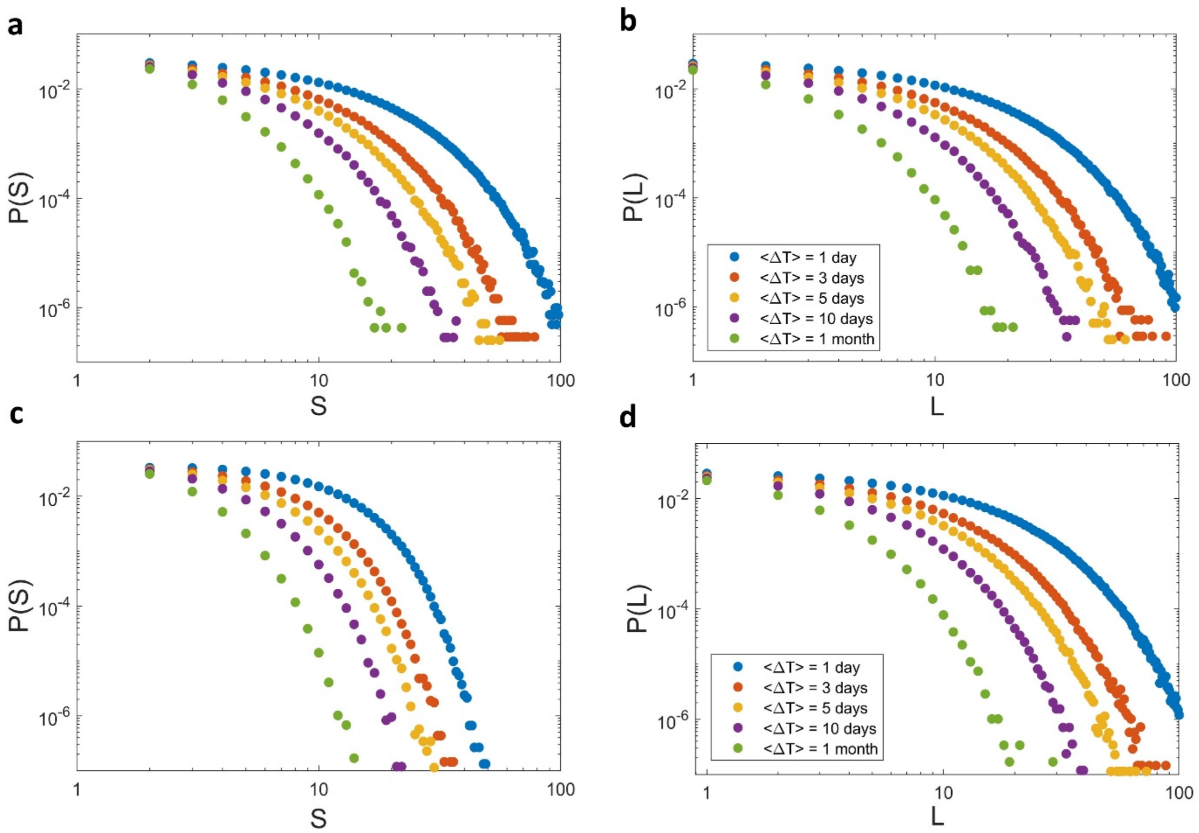

Figure S6. **Illustration of the behavior of infectious-individual mobility on the different relative attractiveness of the locations. (a,c)** The probability distribution of the number of locations visited by an infectious individual, $P(S)$, and **(b,d)** the probability distribution of the number of step lengths among the visited locations of an infectious individual, $P(L)$, for Belgium (above) and Martinique (below).

Characteristics of relative attractiveness

Figure S7a and **S7b** shows the relationship between the population size and the RA in each location for Belgium and Martinique, respectively. We found that the RA is proportional to the population size both in Belgium and Martinique. The populated locations have more attractiveness to attract individuals than locations with a low population density. In addition, the distributions of RA (**Figure S7c**) show that the RA in Martinique is more homogeneous than in Belgium. Interestingly, there are approximately six locations in which their RA is greater than the others both in Belgium and Martinique (**Figure S7d**). The average RA of these locations is 3 times greater in Belgium than in Martinique. It means that individuals in Belgium are likely to be trapped in these locations more than in Martinique, causing individuals in Martinique to have a tendency to visit more locations than in Belgium.

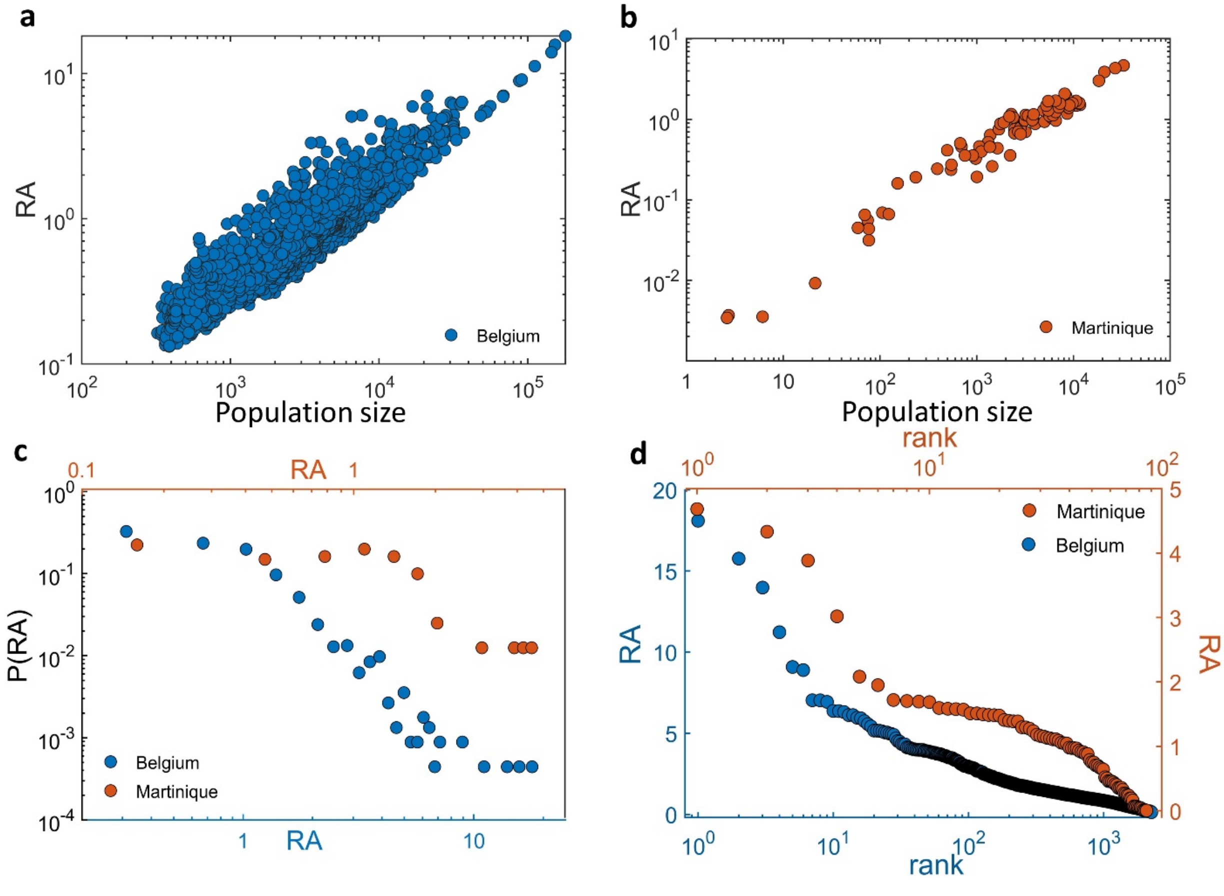

Figure S7. **The relative attractiveness of locations (RA) for Belgium and Martinique**. **(a-b)** The relationship between RA and population size in each location for Belgium and Martinique,

respectively. **(c)** The distributions and **(d)** the ranking values of RA for Belgium (blue) and Martinique (red).

Effects of the population density

In addition, we explored how the population density affects the disease spreading by varying the population ratios; 0.01N, 0.1N, and 1N; $N = 1.1 \times 10^7$, for Belgium and 0.1N, 1N, and 10N; $N = 4 \times 10^5$, for Martinique with $< \Delta T >= 5$ days, as shown in **Figures S8** and **S9**, respectively. We found that the curve of incidence cases is shifted to the right with a higher peak and a narrower width when the population ratio increases, which can be seen in both Belgium (**Figure S8a**) and Martinique (**Figure S9a**). To elucidate the spatial spreading of disease, we computed the contour of time of the first arrival of the infection, T_{fi}, in each location in Belgium (**Figure S8c- S8e**) and Martinique (**Figure S9c- S9e**). Moreover, the relationship between T_{fi} and RA shows that a change in the population density affects the disease transmission in Belgium (**Figure S8b**) more than that in Martinique (**Figure S9b**). All fit parameter values are shown in **Figure S10**.

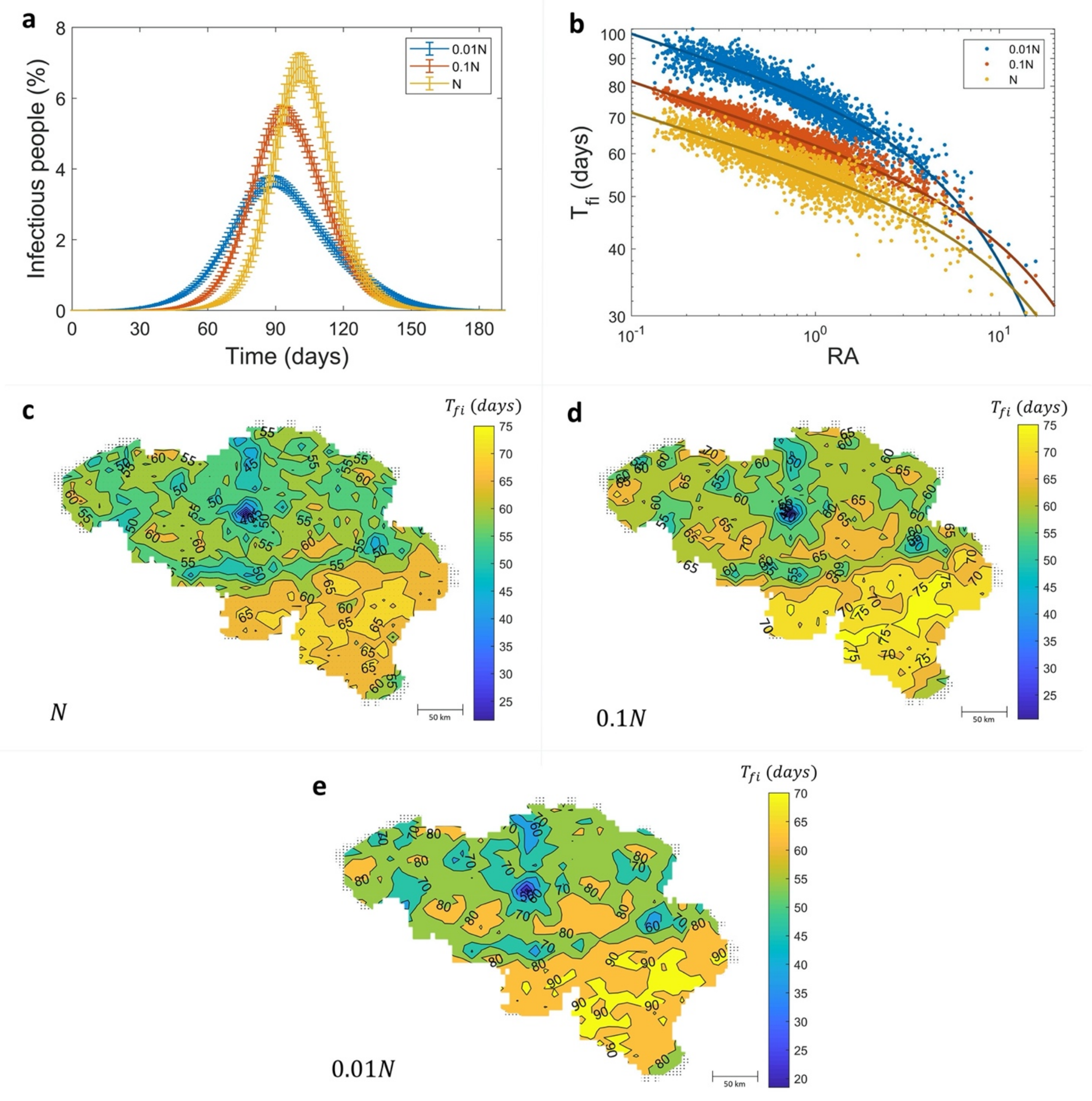

Figure S8. **Effects of population density in Belgium. (a)** The number of infectious people. **(b)** The relationship between RA and T_{fi}. Symbols indicate the results obtained from the model simulations. Lines correspond to the truncated power-law equation. **(c-e)** Contours showing the time of the first arrival of the infection in each location for the country population size of N, $0.1N$, and $0.01N$; $N = 1.1 \times 10^7$, respectively.

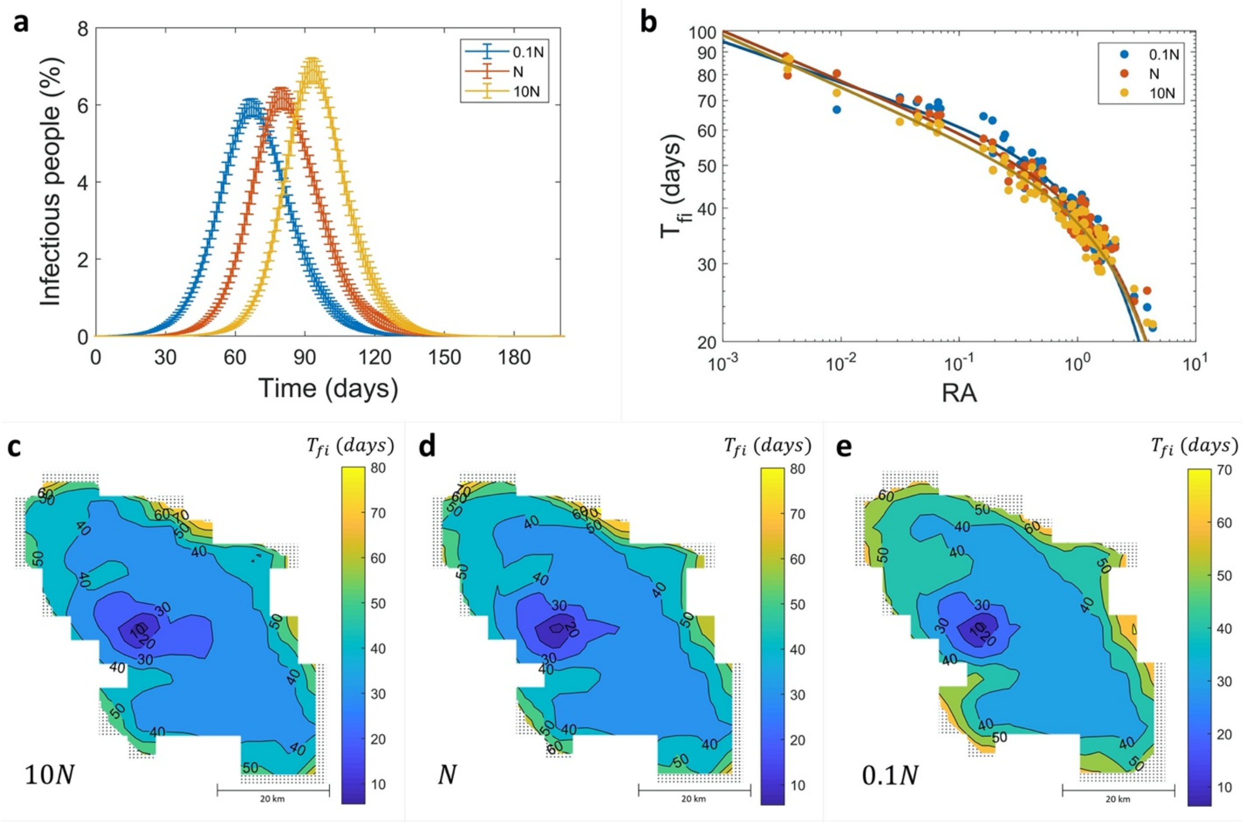

Figure S9. **Effects of population density in Martinique. (a)** The number of infectious people. **(b)** The relationship between RA and T_{fi}. Symbols indicate the results obtained from the model simulations. Lines correspond to the truncated power-law equation. **(c-e)** Contours showing the time of the first arrival of the infection in each location for the country population size of $0.1N$, N, and $10N$; $N = 4 \times 10^5$, respectively.

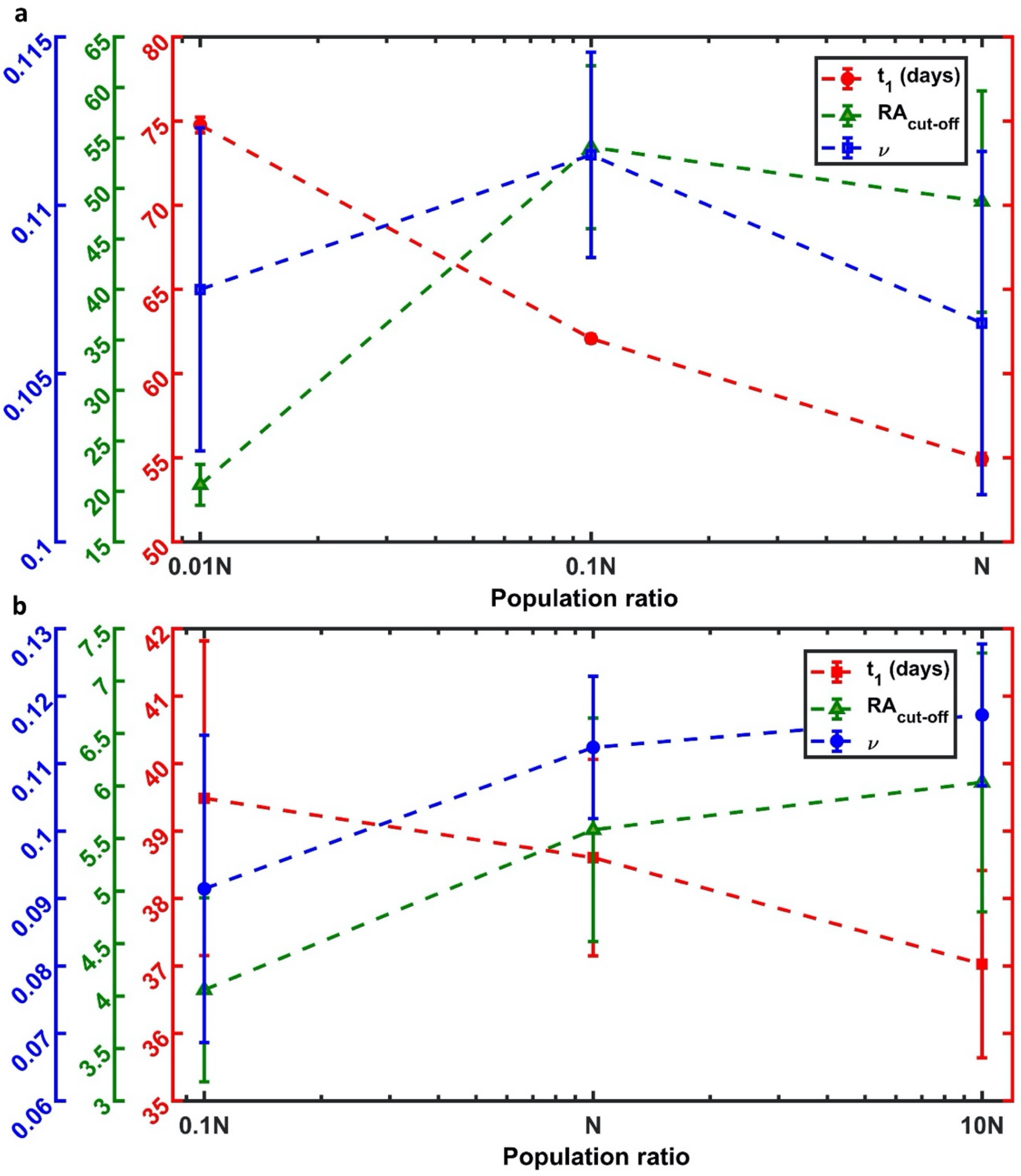

Figure S10. The trend of the fit parameter values. (a-b) t_1, $RA_{cut-off}$, and ν at different population ratio for Belgium; $N = 1.1 \times 10^7$, and for Martinique; $N = 4 \times 10^5$, respectively.

Effects of the waiting times

To investigate how the waiting time of human mobility affects the dynamics of disease transmission, we simulated the model using different values of the average waiting time, $< \Delta T >$; 1, 3, 5, 10, and 30 days, both in Belgium and Martinique. We found that the averaged waiting time of human mobility can affect the profiles of the number of infectious individuals. The epidemic peaks are more shifted from day 78 to day 117 in Belgium (**Figure S11a**) than in Martinique (**Figure S12a**), which is shifted from day 79 to day 85, when $< \Delta T >$ increases from 1 day to 30 days. Moreover, we also measured the epidemic sizes after the end of epidemics. We found that the epidemic size decreases from 74% for $< \Delta T >$ = 1 day to 40% for $< \Delta T >$ = 30 days in Belgium but, in contrast, in Martinique, the increasing in the averaged waiting time does not significantly affect the epidemic size, which stays around 76%. Similarly, the relationship between T_{fi} and RA changes more in Belgium (**Figure S11b**) than in Martinique (**Figure S12b**), when $< \Delta T >$ increases. This effect can be also seen in **Figure S13** showing the trend of the fit parameter values. The contours of T_{fi} for Belgium and Martinique are presented in **Figure S11c-S11g** and **Figure S12c-S12g**, respectively.

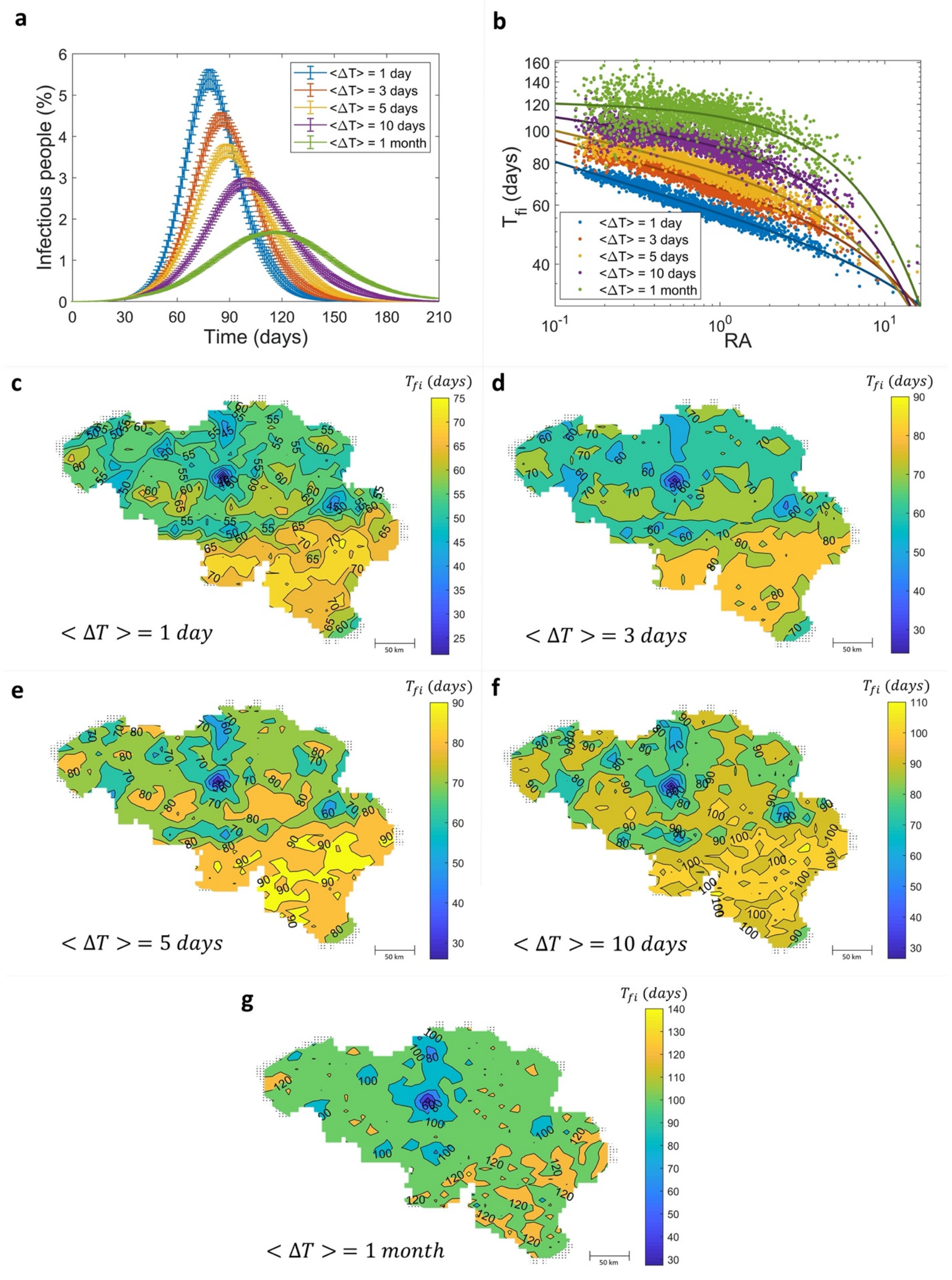

a
Infectious people (%)
Time (days)
<ΔT> = 1 day
<ΔT> = 3 days
<ΔT> = 5 days
<ΔT> = 10 days
<ΔT> = 1 month
b
T_{fi} (days)
RA
<ΔT> = 1 day
<ΔT> = 3 days
<ΔT> = 5 days
<ΔT> = 10 days
<ΔT> = 1 month
c
T_{fi} (days)
$< \Delta T > = 1\ day$
50 km
d
T_{fi} (days)
$< \Delta T > = 3\ days$
50 km
e
T_{fi} (days)
$< \Delta T > = 5\ days$
50 km
f
T_{fi} (days)
$< \Delta T > = 10\ days$
50 km
g
T_{fi} (days)
$< \Delta T > = 1\ month$
50 km

Figure S11. **Effects of waiting time in Belgium. (a)** The number of infectious people. **(b)** The relationship between RA and T_{fi}. Symbols indicate the results obtained from the model simulations. Lines correspond to the truncated power-law equation. **(c-g)** Contours showing the time of the first arrival of the infection in each location for $< \Delta T > = 1, 3, 5, 10,$ and 30 days, respectively.

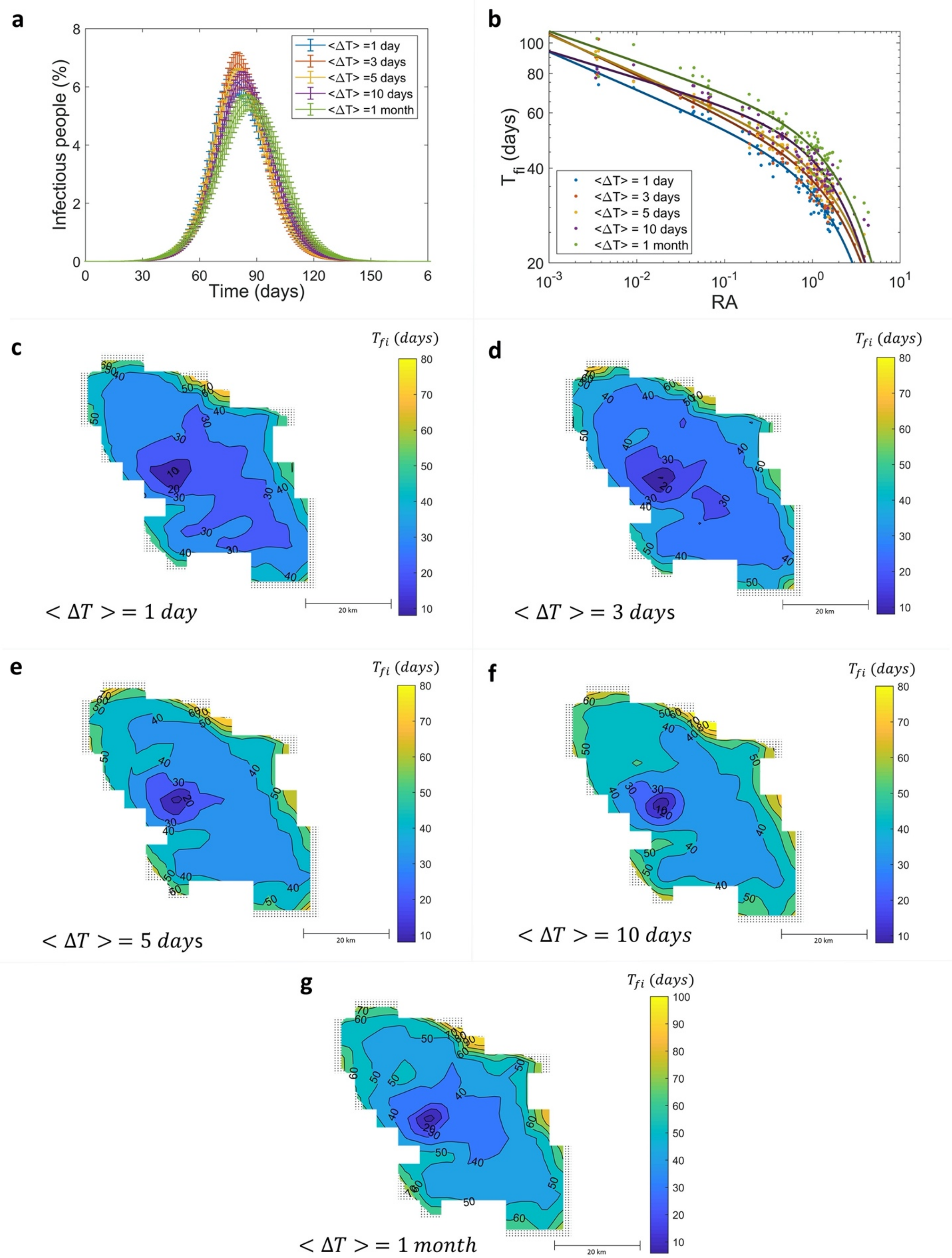

a
Infectious people (%)
Time (days)
<ΔT> =1 day
<ΔT> =3 days
<ΔT> =5 days
<ΔT> =10 days
<ΔT> =1 month
b
T_{fi} (days)
RA
<ΔT> = 1 day
<ΔT> = 3 days
<ΔT> = 5 days
<ΔT> = 10 days
<ΔT> = 1 month
c
T_{fi} (days)
$<\Delta T> = 1\ day$
20 km
d
T_{fi} (days)
$<\Delta T> = 3\ days$
20 km
e
T_{fi} (days)
$<\Delta T> = 5\ days$
20 km
f
T_{fi} (days)
$<\Delta T> = 10\ days$
20 km
g
T_{fi} (days)
$<\Delta T> = 1\ month$
20 km

Figure S12. **Effects of waiting time in Martinique. (a)** The number of infectious people**. (b)** The relationship between RA and T_{fi}. Symbols indicate the results obtained from the model simulations. Lines correspond to the truncated power-law equation. **(c-g)** Contours showing the time of the first arrival of the infection in each location for $< \Delta T > = 1, 3, 5, 10,$ and 30 days, respectively

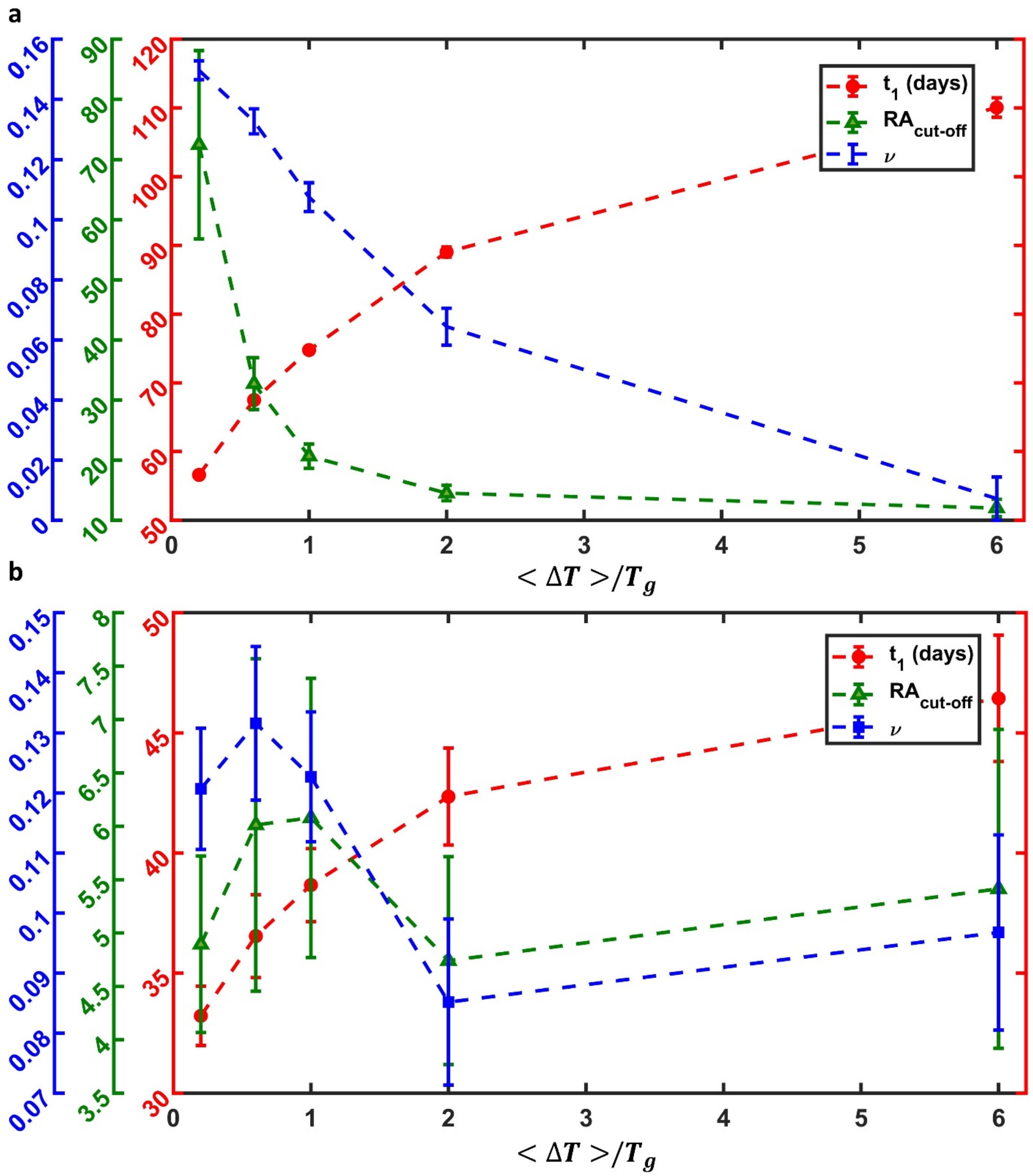

Figure S13. The trend of the fit parameter values. (a-b) t_1, $RA_{cut-off}$, and ν at different $<\Delta T>$, which are $1, 3, 5, 10$ and 30 days; for Belgium and Martinique, respectively.

Effects of restriction on symptomatic infectious individual movement

Figure S14 shows the values of fit parameters of the relationship between T_{fi} and RA shown in **Figure 5** in the main text. When the percentage of symptomatic infectious individual movement restriction (η) increases, the arrival time, t_1, that disease reaches the flat lands increases, and $RA_{cut-off}$ and ν stay in the same trend.

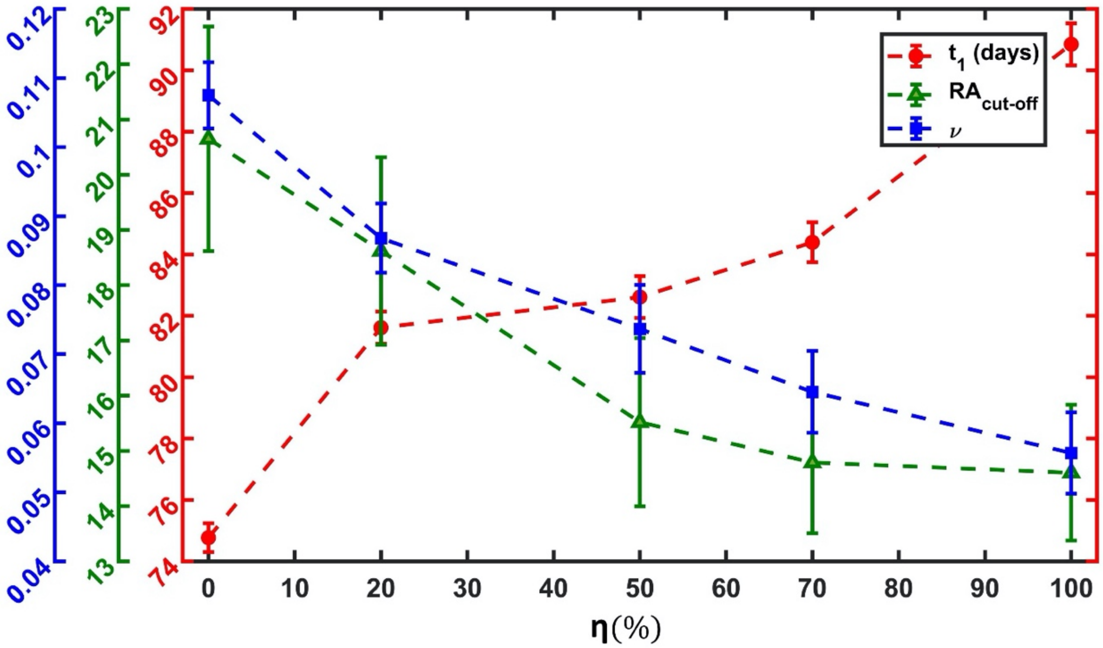

Figure S14. The trend of the fit parameter values. t_1, $RA_{cut-off}$, and ν at different η , which are 0%, 20%, 50% ,70%, and 100 % for Belgium.

References

1. Keeling, M.J. and P. Rohani, *Modeling infectious diseases in humans and animals*. 2011: Princeton University Press.
2. Gillespie, D.T., *Approximate accelerated stochastic simulation of chemically reacting systems.* The Journal of Chemical Physics, 2001. **115**(4): p. 1716-1733.
3. Gillespie, D.T., *Exact stochastic simulation of coupled chemical reactions.* The journal of physical chemistry, 1977. **81**(25): p. 2340-2361.
4. Gillespie, D.T., *A general method for numerically simulating the stochastic time evolution of coupled chemical reactions.* Journal of computational physics, 1976. **22**(4): p. 403-434.
5. Gonzalez, M.C., C.A. Hidalgo, and A.-L. Barabasi, *Understanding individual human mobility patterns.* nature, 2008. **453**(7196): p. 779.
